# Remodeling of the Cardiac Extracellular Matrix Proteome During Chronological and Pathological Aging

**DOI:** 10.1016/j.mcpro.2023.100706

**Published:** 2023-12-21

**Authors:** Deolinda Santinha, Andreia Vilaça, Luís Estronca, Svenja C. Schüler, Catherine Bartoli, Annachiara De Sandre-Giovannoli, Arnaldo Figueiredo, Maximillian Quaas, Tilo Pompe, Alessandro Ori, Lino Ferreira

**Affiliations:** 1 Faculty of Medicine, University of Coimbra, Celas, Coimbra, Portugal; 2 CNC - Center for Neuroscience and Cell Biology, CIBB - Centre for Innovative Biomedicine and Biotechnology, University of Coimbra, Rua Larga, Coimbra, Portugal; 3 CARIM School for Cardiovascular Diseases, Faculty of Health, Medicine and Life Sciences, Maastricht University, Maastricht, Netherlands; 4 Leibniz Institute on Aging, Fritz Lipmann Institute, Jena, Germany; 5 Aix Marseille Univ, INSERM, MMG, U1251, Marseille, France; 6 Molecular genetics laboratory, La Timone children’s hospital, Marseille, France; 7 Serviço de Urologia e Transplantação Renal, Centro Hospitalar Universitário Coimbra EPE, Faculty of Medicine, University of Coimbra, Coimbra, Portugal; 8 Institute of Biochemistry, Faculty of Life Sciences, Leipzig University, Leipzig, Germany

**Keywords:** aging, extracellular matrix, matrisome, proteomic, Hutchinson-Gilford progeria syndrome, cardiac aging, decellularization, lactadherin

## Abstract

Impaired extracellular matrix (ECM) remodeling is a hallmark of many chronic inflammatory disorders that can lead to cellular dysfunction, aging, and disease progression. The ECM of the aged heart and its effects on cardiac cells during chronological and pathological aging are poorly understood across species. For this purpose, we first used mass spectrometry-based proteomics to quantitatively characterize age-related remodeling of the left ventricle (LV) of mice and humans during chronological and pathological (Hutchinson-Gilford progeria syndrome (HGPS)) aging. Of the approximately 300 ECM and ECM-associated proteins quantified (named as Matrisome), we identified 13 proteins that were increased during aging, including lactadherin (MFGE8), collagen VI α6 (COL6A6), vitronectin (VTN) and immunoglobulin heavy constant mu (IGHM), whereas fibulin-5 (FBLN5) was decreased in most of the data sets analyzed. We show that lactadherin accumulates with age in large cardiac blood vessels and when immobilized, triggers phosphorylation of several phosphosites of GSK3B, MAPK isoforms 1, 3, and 14, and MTOR kinases in aortic endothelial cells (ECs). In addition, immobilized lactadherin increased the expression of pro-inflammatory markers associated with an aging phenotype. These results extend our knowledge of the LV proteome remodeling induced by chronological and pathological aging in different species (mouse and human). The lactadherin-triggered changes in the proteome and phosphoproteome of ECs suggest a straight link between ECM component remodeling and the aging process of ECs, which may provide an additional layer to prevent cardiac aging.

## Highlights

• Chronological and pathological aging alters the matrisome of mouse and human myocardium.
• Decellularization enables deeper characterization of age-related alterations in the matrisome.
• Lactadherin is a cardiovascular aging marker that accumulates in large vessels.
• Lactadherin increases the phosphorylation of signaling pathways linked with aging.

## Graphical Abstract

**Figure:**
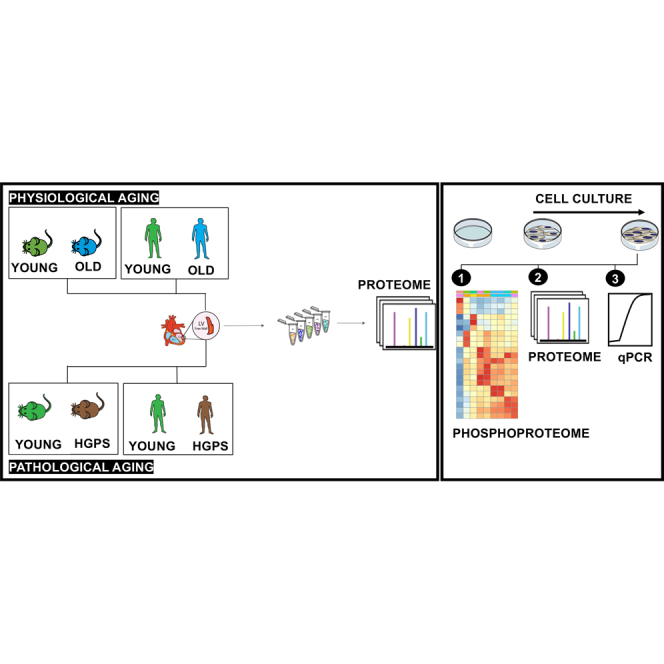

## In Brief

Our study, using recent proteomic methods and strategies to characterize cardiac samples (*e.g.* decellularization), provides new insights into how the cardiac tissue ages, particularly the left ventricle, during chronological and pathological (accelerated) aging. Our results show that the extracellular matrix, secreted by cells, is significantly altered during aging. We further show that lactadherin is a cardiovascular aging marker that accumulates in the vasculature of the left ventricle myocardium and activates age-related signaling pathways in mouse aortic endothelial cells.

Aging is a primary risk factor for the occurrence and development of cardiovascular diseases (CVDs), leading to high morbidity and mortality (1). Chronological aging of the heart is associated with changes at functional, structural, cellular, and molecular levels (2). However, the contribution of non-cellular components such as the extracellular matrix (ECM) and ECM-associated proteins (named matrisome (3)) have been less investigated in the context of aging. The ECM consists of structural and soluble proteins that are involved in various cellular processes such as cell migration, proliferation, cell communication, and differentiation (4, 5). The ECM of the aged heart is characterized by an increase in collagen deposition and its cross-linking (6). Aged cardiac ECM has been shown to promote a senescence phenotype in cardiomyocytes derived from induced pluripotent stem cells (7). However, little is known about the effects of aged ECM on cardiac cells and whether the effects are mediated by the mechanical properties of ECM (as recently shown in the central nervous system (8)) or by its composition (6).

The composition, not just the mechanical properties, of aged ECM may influence its impact on cellular function. Although very little is known in the cardiac context, the decline in fibronectin levels in skeletal muscle leads to the loss of muscle stem cells (9). In addition, expression of SPARC-related modular calcium-binding protein two during muscle aging leads to impairment of muscle stem cells through aberrant activation of integrin/MAPK signalling (10).

Proteomic studies have shown significant differences in cardiac protein composition during aging (11, 12, 13), but few documented changes at the level of ECM (14, 15). This may in part be due to limitations in the detection and quantification of ECM components in highly complex cardiac tissue lysates. To date, it is unclear how aging affects cardiac ECM composition in different animal species and whether chronological and pathological ageing lead to similar changes.

Here, we investigated how chronological and pathological aging (Hutchinson-Gilford progeria syndrome (HGPS)) alters the total proteome and matrisome of the LV myocardium in mice and humans. HGPS is a childhood disease that causes premature aging and early death in children (15, 16). Patients with HGPS often suffer from cardiovascular diseases such as accelerated atherosclerosis, vascular stiffening and calcification, electrocardiographic changes, and LV diastolic dysfunction (17). We performed deep proteomic analyses of LV cardiac muscle from young, old, and HGPS mice as well as human individuals. Our proteomic data enabled us to obtain a deep coverage of the matrisome (>300 proteins) and identify a conserved signature in the old heart. We revealed lactadherin (MFGE8) as a cardiovascular aging marker that accumulates in large vessels during aging. Mechanistically, we show that lactadherin increases the phosphorylation of glycogen synthase kinase-3 beta (GSK3B) and MTOR kinases targets, decreases the phosphorylation of CDK targets, and promotes the expression of pro-inflammatory effectors in endothelial cells.

## Experimental Procedures

An extended description of methods is provided in the Supplementary Material.

### Mice

This study was conducted in accordance with the guidelines of Directive 2010/63/EU of the European Parliament on the protection of animals used for scientific purposes and was approved by the local Ethical Committees. All mice were euthanized with 0.5 ml/min CO_2_ inhalation for 5 min, then sacrificed and the left ventricles isolated. Five male C57BL/6 Wild-type mice aged 20 months and 3 months were obtained from in-house breeding at the Leibniz Institute on Aging - Fritz Lipmann Institute (FLI) and at UC -Biotech (University of Coimbra), respectively. Young male mice (3 months old) were also purchased from Janvier. Animals were housed in a dedicated pathogen-free animal facility with a 12-h light-dark cycle and fed a standard diet ad libitum. Formalin-fixed and paraffin-embedded (FFPE) mouse heart samples from two male and three female Lmna^G609G/G609G^ mice (Knock-in mice carrying the HGPS mutation described elsewhere (18)) (10 weeks) and age-matched Wild-type (WT) were kindly provided by Annachiara De Sandre-Giovannoli.

### Human Left Ventricular Postmortem Tissues

All human hearts included in this study were not suitable for transplantation according to tissue bank regulations. Postmortem human LV samples were obtained from Coimbra Hospital University Centre (CHUC, Coimbra) from four elderly donors (65–70 years old) and one young donor (31 years old). Additional human FFPE LV samples from young donors (20–21 years old) were kindly provided by the Centre for Heart Lung Innovation Providence Health Care/University of British Columbia. Samples of cardiac tissue were obtained when no cardiac involvement was indicated in complications leading to death. Clinical characteristics of the elderly and young donors are shown in supplemental Table S4. The Progeria Research Foundation donated FFPE tissue sections from LV tissue from two donors with Progeria syndrome (10 and 14 years old). According to the Common Rule and National Law *No. 12/93, de 22 de Abril*, deceased individuals do not meet the definition of a human subject, so informed consent is not required for the use of postmortem samples. However, the investigations performed were approved by the local ethics committee of the Faculty of Medicine of the University of Coimbra (CE -022/2017) and were in accordance with institutional guidelines and the principles of the Declaration of Helsinki.

### Cells

Mouse aortic endothelial cells (MAECs) were purchased from PELOBiotec (CellBiologics, C57–6052)). MAECs were grown in Complete Mouse Endothelial Cell Medium supplemented with VEGF, ECGS, heparin, EGF, hydrocortisone, L-glutamine, and 5% of fetal bovine serum (CellBiologics, M1168) according to the manufacturer’s instructions. Cells were cultured in a 37 °C incubator with 5% CO_2_ and 95% humidity.

### Decellularization of the Mouse Left Ventricle

Similar LV portions (6–9 mg) from five young and five old animals were decellularized following the protocol adopted from Silva *et al*. 2016 (19). The decellularized LV tissue portions were stored at −80 °C until further use. For a schematic representation of the decellularization protocol, see Figure 3*A*.

**Fig. 1:**
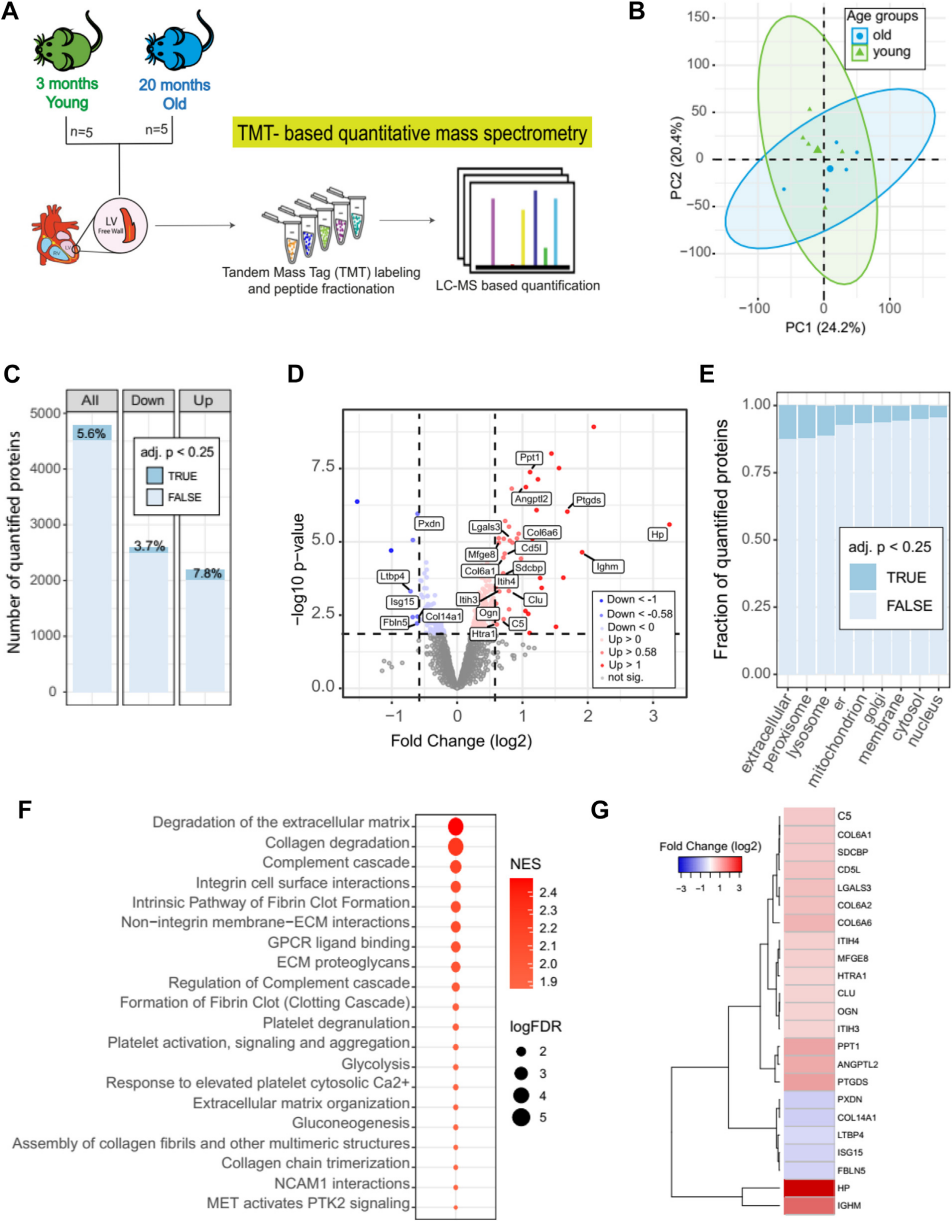
**Age-related changes in the LV proteome of mouse heart tissue.***A*, schematic workflow of proteomics experiments. The proteome of left ventricular heart tissue from 20-month-old mice (old) was compared to the proteome of young mice (3 months). Both age groups were analyzed by TMT-based quantitative mass spectrometry. Five male C57BL/6 mice were used for each age group. *B*, PCA plot shows the correlation of age-related alterations. Ellipses represent 95% confidence intervals. The percentage of variance is shown for each principal component (PC). *C*, number of all quantified protein groups and up- and down-altered proteins. The percentage of significantly affected proteins indicated in *dark blue* (adj. *p* < 0.25) was calculated based on the total number of proteins in each column. *D*, Volcano plot showing the significant up- (*red*) and down-regulated (*blue*) proteins (adj. *p* value <0.25 and absolute log2 fold change >0.58) comparing old vs young LV proteome. Proteins not affected are shown in *grey.E*, compartmental analysis displaying the proportion of significantly age-affected proteins (adj. *p* < 0.25) for each cellular compartment. In the plot, “er” represents the endoplasmic reticulum and “extracellular” includes ECM and ECM-associated proteins. *F*, gene set enrichment analysis (GSEA) shows the 20 most affected pathways during ageing. GSEA was performed using WebGestalt (64). NES: Normalized Enrichment Score; FDR: False Discovery Rate. *G*, heatmap exhibiting all age-altered ECM and ECM-associated proteins (adj. *p* < 0.25) and with an absolute fold change (log2) greater than 0.5.

**Fig. 2:**
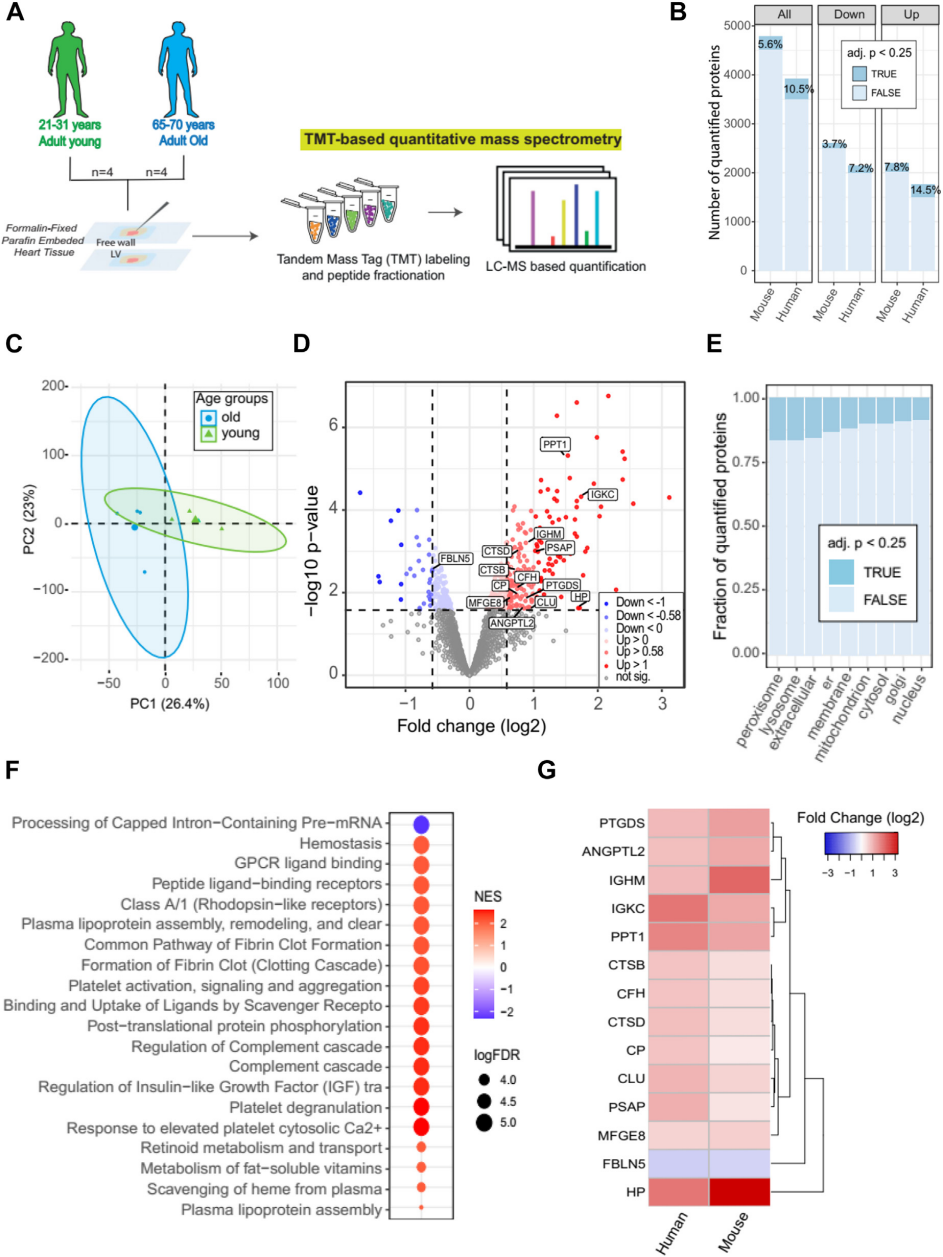
**Effects of aging on human LV tissue.***A*, schematic workflow of proteomics experiments using formalin-fixed paraffin-embedded (FFPE) LV (free wall) tissue from human heart. FFPE left ventricular tissue from young (25–31 years old) and old (65–70 years old) adults was analysed by TMT-based quantitative mass spectrometry. *B*, number of all and differentially (*up and down*-regulated proteins) quantified protein groups in old and young human LV tissues and in mice (data previously shown) for comparison of both data sets. The percentage of significantly affected proteins (adj. *p* < 0.25) is indicated in *dark blue.C*, PCA plot showing correlation of age-related alterations. Ellipses represent 95% confidence intervals. The percentage of variance is indicated for each principal component (PC). *D*, volcano plot displaying the significant up- (*red*) and downregulated (*blue*) proteins (adj. *p* value <0.25 and absolute log2 fold change >0.58) comparing *old versus young* human LV proteome. Proteins not affected are shown in *grey*. *E*, compartmental analysis displaying the proportion of significantly age-affected proteins (adj. *p* < 0.25) for each cellular compartment. In the plot, “er” represents the endoplasmic reticulum and “extracellular” includes ECM and ECM-associated proteins". *F*, gene set enrichment analysis (GSEA) indicating the 20 most affected pathways during ageing. GSEA was performed using WebGestalt (64). *G*, heatmap displaying all common age-related changes in ECM and ECM-associated proteins (adj. *p* < 0.25) and with an absolute fold change (log2) >0.5 in human and mouse LV tissues.

**Fig. 3:**
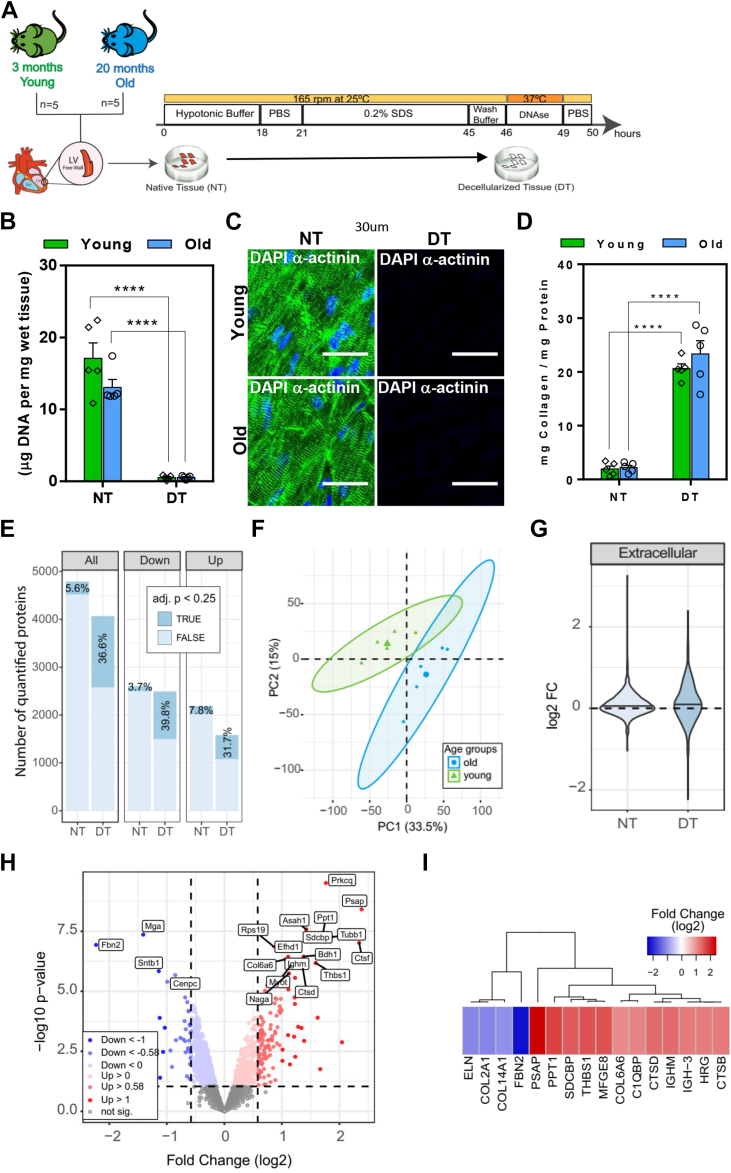
**Age-related changes in the proteome of decellularized tissue of the LV mice**. *A*, schematic workflow of the decellularization approach. Multiple portions of native LV tissue (NT) from five young and five old mice were decellularized (DT) according to the decellularization protocol schematically shown here. *B*, DNA quantification of tissue before, native tissue (NT), and after decellularization, decellularized tissue (DT) of young and old mouse hearts. *C*, immunofluorescence staining for α-actinin (*green*) and DNA by DAPI (*blue*)) of NT and DT from young and old mouse ventricular tissues. Scale bar, 30 μm. *D*, biochemical ratio of mg collagen per mg of total protein quantified in NT and DT from young and old LV mice. *E*, number of all and differentially (*up and down*-altered) quantified protein groups in young and old DT. Data from NT (Fig. 1) were added to facilitate the comparison with DT. The percentage of significantly affected proteins (adj. *p*< 0.25) is indicated in *dark blue.F*, PCA plot showing the correlation of age-related alterations in the proteome of DT. Ellipses represent 95% confidence intervals. Percentage of variance is indicated for each principal component (PC). *G*, Violin plot showing the fold change variation (FC, (log2)) for all quantified ECM proteins in NT and DT. *H*, volcano plot showing the significant up (*red*) and down (*blue*) - altered proteins (adj. *p* value <0.25 and absolute log2 fold change >0.58) comparing *old versus young* DT. Proteins not affected are shown in *grey.I*, heatmap displaying the age-altered proteins (adj. *p* < 0.25) and with an absolute fold change (log2) >1 in DT. In figures *A* and *D*, ∗∗∗∗*p* < 0.0001; ordinary two-way ANOVA.

### Biochemical Quantification of dsDNA, Total Proteins, Collagens, and sGAGs in Native and Decellularized LV Tissue

Double-stranded deoxyribonucleic acid (dsDNA) and major ECM components were quantified from decellularized (DT) and compared with native (NT) LV mouse heart tissue. The dsDNA content was determined using the DNA Quantitation Kit (Sigma-Aldrich, DNAQF) according to the manufacturer’s instructions. The quantity of protein, collagen, and sulfated glycosaminoglycans (sGAG) was quantified using BCA assay (Thermo Scientific, PI23225), QuickZyme Total Collagen assay (Quickzyme Biosciences, QZBTOTCOL1), and Glycosaminoglycan assay Blyscan (Biocolor, B1000), respectively, according to the manufacturer’s instructions. Data were expressed as measured component mass normalized to tissue wet weight or protein content.

### Sample Preparation for MS Analysis

Tissue lysates (50 μg) (see the lysis method in the supplemental Data File) were reduced and then alkylated. Proteins were precipitated, centrifuged and the pellet was washed twice. The pellets were air-dried before adding the appropriate volume of the digestion buffer. Protein digestion was started by adding LysC (Wako Chemical GmbH, 125–05,061), samples were then diluted 1:1 with MilliQ water, and trypsin (Promega, V5111) was added to complete digestion overnight. Digested samples were acidified and then desalted. Eluates were dried in a speed vacuum centrifuge and then dissolved at a concentration of 1 μg/μl in a reconstitution buffer. The reconstituted peptides were used for TMT labeling (supplied in the supplemental Data File).

### Protein Solubilization of FFPE Mouse and Human Samples

Tissue sections were deparaffinized and then were gently scraped using a scalpel and collected into PCR tubes. After protein solubilization (detailed method in the supplemental Data File), the cysteine residues were alkylated. These proteins were then precipitated and after removal of the supernatant, precipitates were washed twice. Pellets were further digested, acidified, and desalted as described in Sample Preparation for MS Analysis.

### RT-qPCR

Total RNA was extracted from the left ventricular tissue of young and old mice and from MAECs using the miRNeasy Micro Kit (Qiagen, 217,084) or the RNeasy Plus Micro kit (QIAGEN, 74,034), respectively. Real-time quantitative PCR (RT-qPCR) of 5 ng RNA used for cDNA synthesis was performed using NZYSpeedy qPCR Green Master Mix (NZYtech, MB224) according to the manufacturer’s instructions, and detection was carried out in the CFX Connect Real-Time PCR system (Bio-Rad).

### Immunofluorescence of Young and Old LV Mouse Heart Tissue

Sections of FFPE mouse LV tissue were cut, mounted on a glass slide, and then deparaffinized. The mouse and human tissue sections underwent heat-mediated antigen retrieval and were blocked after permeabilization. Then, the tissue sections were incubated overnight with the primary antibody. Finally, after washing three times, the sections were incubated with the respective secondary antibody After washing, nuclei were stained with DAPI (2 μg/ml in PBS) (Sigma, D9542) at RT for 5 min followed by three washing steps with PBS and mounting in Mounting Medium. Stained cryosections were observed using a high-content fluorescence microscope (IN Cell 2200, GE Healthcare) or an LSM 710 point-scan confocal laser microscope (Zeiss) with a 40 × oil immersion objective.

### Phosphoproteomic Analysis

MAECs were grown according to the experimental protocol detailed in the supplemental Data File. Cells were scraped, the cell suspension was centrifuged, and pellets were stored at −20 °C until further use. Pellets were lysed in RIPA buffer and were precipitated, digested to peptides and desalted as described in Sample Preparation for MS Analysis. Phosphorylated peptides were enriched with Fe(III)-NTA cartridges (Agilent Technologies G 5496–60085) using the AssayMAP Bravo Platform (Agilent Technologies), as described in Post *et al*. 2017 (20).

### Experimental Design and Statistical Rationale

For the heart proteome analyses, the left ventricular heart tissue of young (n = 5) and aged (n = 5) C57BL/6 mice was used for quantitative mass spectrometry. The proteins were considered significantly altered with an adjusted *p* value <0.25 and absolute log2 fold change >0.58.

For the effect of aging on human LV, the left ventricular heart tissue of young (n = 4) and aged (n = 4) individuals were analyzed by quantitative mass spectrometry. The proteins were considered significantly altered with an adjusted *p* value <0.25 and absolute log2 fold change >0.58, comparing old versus young human LV proteome.

For the age-related changes in the proteome of decellularized tissue of the LV mice, five animals were used in each age group. The proteins were considered significantly altered with an adjusted *p* value <0.25 and with an absolute fold change (log2) >1. An ordinary two-way ANOVA was used to determine the statistically significant differences.

For the impact of HGPS on cardiac tissue, left ventricular tissue from young adults and two individuals diagnosed with HGPS was analyzed by TMT-based quantitative mass spectrometry, while for mice cardiac tissue, five animals were used in each of the three experimental groups (Wild-type, progeroid and HGPS). The proteins were considered significantly altered with an adjusted *p* value <0.25 comparing in mice the progeroid phenotype with the Wild-type and in humans comparing young *versus* old.

For the quantification of lactadherin in LV tissues, three animals per group were used to quantify the percentage of lactadherin measured in the largest to smallest blood vessels of young and old mouse LV tissue, an average of 30 images for each section, two sections for each animal were measured. An unpaired *t* test was used to determine the statistically significant differences.

For the quantification of the localization of lactadherin in blood vessels, three animals were used per group (young and old), and an average of 10 hit areas per section were analyzed, two sections for each animal. A two-tailed unpaired student’s *t* test was used to determine the statistically significant differences.

## Results

### Chronological Aging in Mice and Humans Primarily Affects the Matrisome of the Left Ventricular Cardiac Tissue

The composition and remodeling of the LV during aging was investigated using a mass spectrometry (MS)-based proteomics strategy. We selected the LV because it undergoes important functional remodeling during cardiac aging, including diastolic dysfunction and left ventricular hypertrophy (2). To identify age-related changes in the LV proteome of mouse cardiac tissue, we compared two different age groups of wild-type C57BL/6 mice, young adults ((y), 3 months old, n = 5) and old ((o), 20 months old, n = 5) (supplemental Table S1). For each animal, we obtained a quantitative proteome profile using Tandem Mass Tags (TMT)-based quantitative MS after peptide fractionation at high pH to maximize proteome coverage (Fig. 1*A*) (10). This approach quantified a total of approximately 5000 protein groups across all ages using at least two unique proteotypic peptides, overcoming the limitations in proteome coverage observed in previous studies of cardiac tissue during normal aging (11, 12, 21, 22) (supplemental Fig. S1*A*). The proteomic measurements were highly reproducible, as shown by the low coefficient of variation within both experimental groups (*i.e.*, young vs. old) (supplemental Fig. S1*B*). Although principal component analysis (PCA) showed no obvious separation between old and young proteomes (Fig. 1*B*), 5.6% of proteins had different abundance between young and old LVs (Fig. 1*C*). In the old heart, the percentage of significantly upregulated proteins (adj.*p* < 0.25) was higher than that of downregulated ones (Fig. 1, *C* and *D* and supplemental Table S2). Some of our results are consistent with previous reports of aged LV mice that showed strong deposition of collagen VI (14). Next, we examined which cellular compartments were more affected by aging, and we found that the extracellular compartment (which includes ECM and ECM-associated proteins), peroxisomes, and lysosomes had the highest proportion of affected proteins (>10%, Fig. 1*E*). Next, we used Gene Set Enrichment Analysis (GSEA) to investigate which molecular networks and signaling pathways were altered in old hearts compared with young hearts (Fig. 1*F*). ECM-related signaling pathways such as ECM degradation, followed by integrin-cell surface interactions, non-integrin membrane-ECM interactions and ECM proteoglycans showed the highest enrichment (supplemental Table S3). Out of the 23 ECM and ECM-associated proteins that were significantly affected, 18 showed increased abundance with age, indicating a general accumulation of ECM components in the old mouse LV (Fig. 1*G* and supplemental Fig. S1*C*).

We next extended the analysis to human heart tissue by analyzing the proteome of LV samples from four young donors aged 21 to 31 years (young group (y)) and four donors aged 65 to 70 years (old group (o)) (supplemental Table S4) (Fig. 2*A*). Because of the limited availability of these samples, we used formalin-fixed and paraffin-embedded (FFPE) samples to quantitatively analyze the human LV proteome. For this purpose, we used a recently developed protocol using TMT-based MS quantification (23). This protocol provided protein yields that were consistent with the analysis of fresh frozen material (24). Using this approach, we quantified ∼4000 protein groups, of which 10.5% were significantly age-affected (adj.*p* < 0.25), and as observed in the mouse data, the percentage of upregulated proteins was higher than that of downregulated proteins (Fig. 2*B* and supplemental Table S5). Although the coefficient of variation was slightly different between young and old, the reproducibility of the data was relatively high in both groups (supplemental Fig. S1*D*). Importantly, PCA showed a clear separation between the proteomic profiles of the old and young groups (Fig. 2*C*). We next examined which cellular compartment was most affected by aging in humans LV. Similar to mice, peroxisomes, lysosomes, and the extracellular (which includes ECM and ECM-associated proteins) compartment had the highest percentage of proteins affected by aging (Fig. 2*E*). Using GSEA, we also identified the signaling pathways that are most altered by chronological aging in humans LV (Fig. 2*F*). Importantly, we identified a subset of ECM and ECM-associated proteins that were consistently affected by aging in LV of mice and humans (Fig. 2*G*).

Overall, our results in mouse and human LVs showed that: (i) the percentage of significantly up-regulated proteins was higher than that of down-regulated ones and (ii) the ECM and ECM-associated proteins were the most affected by ageing.

### Decellularization Enables Deeper Characterization of Age-Related Alterations in the Matrisome of Cardiac Tissue

To obtain higher sensitivity regarding matrisome composition, we applied a decellularization procedure based on SDS to native tissue (NT) of mice LV from both age groups (old and young mice) to obtain a decellularized tissue (DT) (Fig. 3*A*). Decellularization strategies can be used to enrich the content of ECM proteins (14, 25) in order to have a deeper knowledge about their structure (19) and composition (26). Prior to proteome-based MS analysis, the efficiency of the decellularization process was confirmed by a reduction in cellular DNA content (Fig. 3*B* and supplemental Fig. S2*A*, HE), the absence of several cellular components (nuclei and α-actinin) (Fig. 3*C*), and the maintenance of the integrity of the ECM structure in the DT compared with the NT groups (supplemental Fig. S2*A* (PSR) and supplemental Fig. S2*B*). The enrichment of ECM and ECM-associated proteins in DT was evidenced by the higher ratio of collagen per mg of total protein (Fig. 3*D*). The microstructure of DT was examined by scanning electron microscopy which showed that both acellular tissues had a compact fiber network (supplemental Fig. S2*C*). However, the elastic modulus showed a lower stiffness of DT compared to NT (supplemental Fig. S2*D*). Quantitative analysis revealed a higher level of the major components of the ECM, collagen, and sulfated glycosaminoglycan's (sGAGs), and lower total protein content in DT compared with NT (supplemental Fig. S2, *E*–G).

Next, we analyzed the proteome composition of DT in old and young groups and compared them with the results of NT (data previously shown in Fig. 1). In DT, a total of about 4000 protein groups were reproducibly quantified using at least two unique proteotypic peptides (Fig. 3*E*, supplemental Fig. S2*H*, and supplemental Table S6). DT showed a higher number of significantly altered proteins (*adj.p* < 0.25) compared to NT (36.6% *versus* 5.6%, respectively; Fig. 3*E*). PCA showed a clear separation between age groups (Fig. 3*F*). The higher sensitivity of DT for detecting age-related changes in ECM proteins was supported by the fact that ECM and ECM-associated proteins showed overall larger fold changes (log2) in DT compared to NT (Fig. 3*G*). ECM and ECM-associated proteins that were increased in the old compared to the young group included prosaposin (PSAP), palmitoyl-protein thioesterase 1 (PPT-1), syndecan binding protein (SDCBP), thrombospondin-1 (THBS1), and lactadherin (MFGE8) (Fig. 3, *H* and *I*). Importantly, we detected a greater number of significantly age-affected ECM and ECM-associated proteins in DT (supplemental Fig. S2*I*), including laminin subunit alpha-2 (LAMA2), stromal cell-derived factor 1 (SDF-1), SPARC-related modular calcium-binding protein 2 (SMOC-2) and dystroglycan (DAG1), which were not observed in whole-cell lysates (supplemental Fig. S2*J*), highlighting the importance of decellularization in revealing age-related changes in the matrisome of LV mouse heart tissue. However, the matrisome quantified in both, NT and DT, showed similar regulation by aging (supplemental Fig. S2*K*).

Taken together, the proteomic results of the decellularized ECM showed a higher number of significantly altered proteins in the aged LV relative to NT, some of them not being detected in the NT experimental group.

### Pathological Aging/HGPS Primarily Affects the Nucleus and Mitochondrial Cell Compartment in the Left Ventricle Proteome

**Fig. 4:**
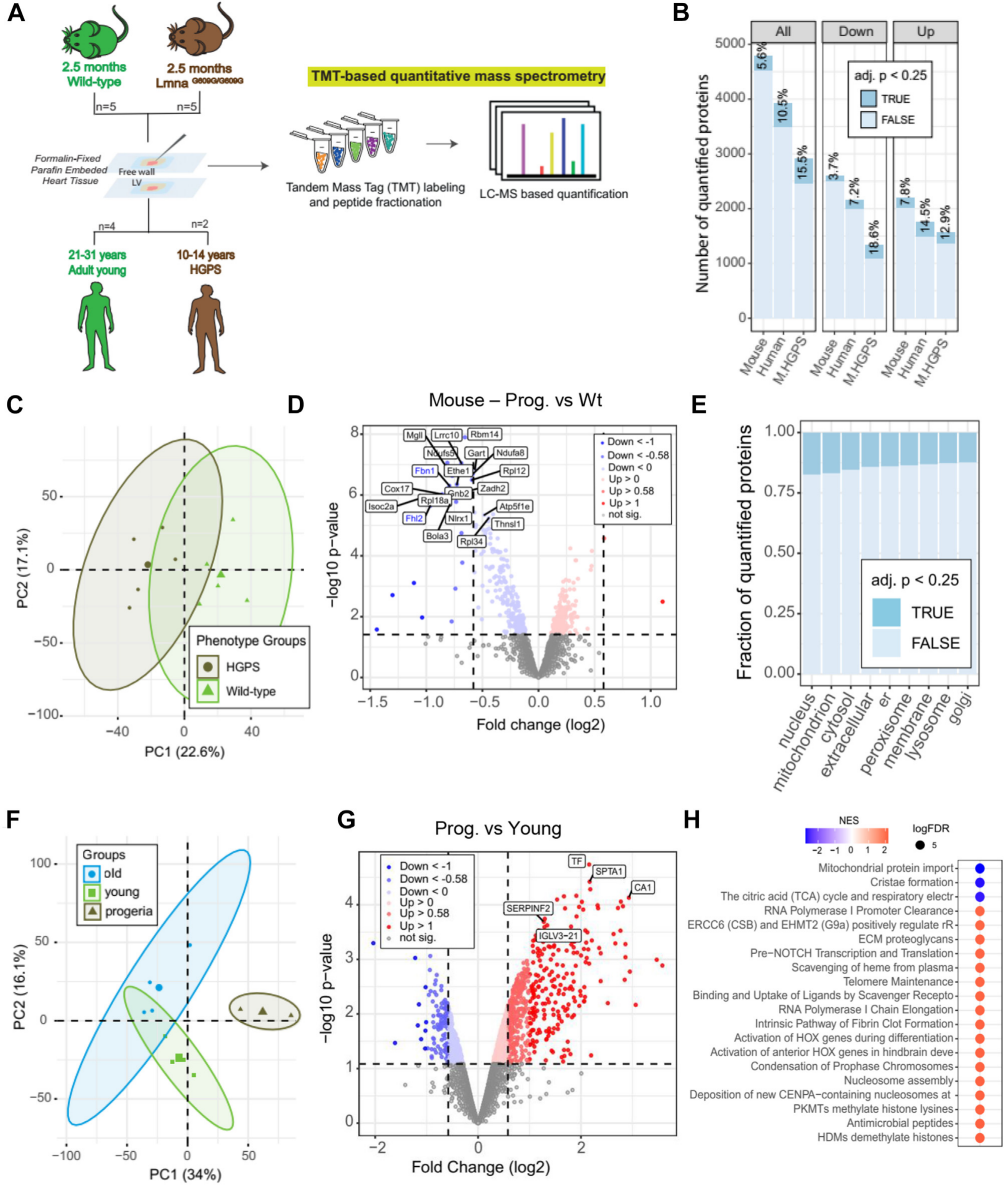
**Impact of HGPS on cardiac tissue in mice and humans.***A*, schematic workflow of proteomic experiments using formalin-fixed paraffin-embedded (FFPE) left ventricles (free wall) of mice and humans. The proteome of LV tissue from age-matched (i) Wild-type and progeroid mice (Lmna^G609G/G609G^) (2.5 months old) and (ii) FFPE left ventricular tissue from young adults (25–31 years old) and two individuals diagnosed with HGPS (10 and 14 years old) was analysed by TMT-based quantitative mass spectrometry. Five male C57BL/6 mice were used for each group, Wild-type and progeroid (Lmna ^G609G/G609G^). Four young male donors were used for the young group and one female and one male donor for the HGPS group. *B*, number of all and differentially (up and down-altered) quantified protein groups in each data set: mouse progeroid (M.HGPS) group (Wild-type vs. progeroid mice) and were added for comparison with previous data sets from mice and humans comparing *young versus old*. The percentage of significantly affected proteins (adj. *p* < 0.25) is indicated in *dark blue*. *C*, PCA plot showing the correlation of phenotype-related changes in the mouse proteome (mouse HGPS and Wild-type). *D*, volcano plot showing the significantly up (*red*) and down (*blue*) altered proteins (adj. *p* value <0.25 and absolute log2 fold change >0.58) comparing the progeroid phenotype with the Wild-type. Proteins not affected are shown in *grey.E*, compartmental analysis displaying the proportion of significantly altered proteins (adj. *p* < 0.25) by HGPS for each cellular compartment. In the plot, “er” means endoplasmic reticulum and “extracellular” represents ECM and ECM-associated proteins”. *F*, PCA plot showing the correlation among age-related alterations (*old and young*) and HGPS in human LV proteome. *G*, volcano plot displaying the dispersion of all quantified proteins according to their log2 fold changes and statistical significance (adj. *p* < 0.25) when comparing HGPS with young human LV tissue. *H*, gene set enrichment analysis (GSEA) display of the 20 most affected pathways during pathological aging in humans. GSEA was performed using WebGestalt (64). In the PCAs, the ellipses represent 95% confidence intervals. For each principal component (PC), the percentage of variance is indicated.

To investigate the differences in cardiac tissue between pathological/HGPS and chronological aging, we used mice carrying a homozygous point mutation of the Lamin A/C (*LMNA*) gene, (the *Lmna*^*G609G/G609G*^ mice), characterized by abnormal accumulation of the mutated protein “progerin” and a premature aging phenotype. Among existing mouse models for HGPS, this knock-in mouse model recapitulates most of the cardiovascular aspects of this rare disease (18). We analyzed by proteomics LV from five C57BL/6 10-week-old Lmna^G609G/G609G^ mice (HGPS group) and five age-matched LV C57BL/6 Wild-type mice (Wt group) (supplemental Table S7) (Fig. 4*A*). Approximately 3000 proteins (M.HGPS) were quantified (supplemental Table S8). The low coefficient of variation within the two experimental groups (*i.e.*, WT vs. HGPS) showed high reproducibility of proteomic measurements (supplemental Fig. S3*A*). A high percentage (15.5%) of altered proteins (*adj.p* < 0.25) (Fig. 4*B*) was observed between the HGPS and WT groups, consistent with a clear separation of proteomic profiles observed by PCA (Fig. 4*C*). Interestingly, the proportion of downregulated proteins in the HGPS group was higher than that of up-regulated proteins in the Wt. group (18.6% vs. 12.9%, Fig. 4, *B* and *D*), contrasting with what has been observed during chronological aging in both mice and humans. A significant proportion of the differentially abundant proteins in HGPS belonged to the nuclear and mitochondrial compartments (Fig. 4*E*), consistent with previously reported changes in energy metabolism and mitochondrial dysfunction in progeroid mice (13, 27).

We then extended the proteomic analysis to left ventricular cardiac tissue from two humans with HGPS (Progeria group (P)) (supplemental Table S9) (Fig. 4*A*). The reproducibility of the experiments is shown in supplemental Fig. S3*B*. PCA showed a strong separation between the proteomic profiles of healthy donors from both age groups (*i.e.*, young and old) and progeria (Fig. 4*F*). The volcano plot showed a significant number of upregulated proteins in progeria compared with young donors, including serotransferrin (TF), spectrin alpha chain (SPTA1), insulin-like growth factor-binding protein complex acid labile subunit (IGFALS), and others (Fig. 4*G*). Using GSEA, we identified three negatively regulated categories related to mitochondrial mechanisms and several positively altered categories related to nuclear and extracellular mechanisms. We compared the LV proteomic changes induced by chronological and pathological aging in mice and humans. This analysis showed that the proteomic changes induced by chronological aging and HGPS were largely uncorrelated in mice (supplemental Fig. S3*C* and supplemental Table S10). However, in humans, a subset of proteins was identified being consistently elevated (supplemental Fig. S3*D*).

### Physiological Cardiac Aging Leads to Accumulation of Lactadherin

**Fig. 5:**
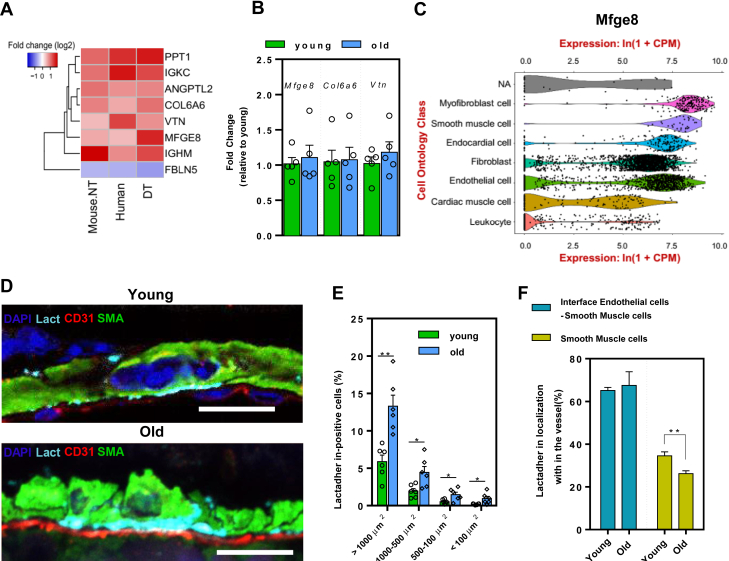
**Validation of proteomic data and localization of lactadherin in LV tissue.***A*, heatmap showing the absolute log two fold change (FC) higher than 0.5 for the eight most statistically affected ECM proteins (*p* < 0.05 and absolute log2 FC > 0.5) and in three of four analysed proteomic datasets, in native mouse (NT), human NT and decellularized mouse LV tissues (DT). *B*, gene expression analysis by RT-qPCR for three of these ECM proteins selected from (a), in LV isolated from 3 months old (*young*) and 20 months old (*old*) C57BL/6 mice. The levels were normalized to the average expression level of the young mice. Results are Average ±SEM, n = 5, per group. *C*, Violin plot showing the expression levels of Mfge8 (lactadherin) in the heart based on single-cell data from Tabula Muris– FACS (30). *D*, representative images of immunofluorescence staining for lactadherin (*Lact*) (*cyan*), CD31 (*red*), smooth muscle actin (SMA) (*green*) and DAPI (*blu*e) from LV sections of 3 months (*young*) and 22 months old (*old*) mice. Scale bar: 10 μm. *E*, bar plot shows the average of the percentage of lactadherin measured in the largest (>1000 μm^2^) to smallest (<100 μm^2^) blood vessels of young and old mouse LV tissue. Results are Average ± SEM, n = 3, per group. An average of 30 images for each section, two sections for each n were measured; ∗∗*p* < 0.01; ∗*p* < 0.05; Unpaired *t* test. *F*, bar plot showing localization of lactadherin in blood vessels. n = 3, per group (*young and old*). An average of 10 hit areas per section were analysed, two sections for each n. ∗∗*p* < 0.01; two-tailed un-paired student’s *t* test.

We next compared the effects of chronological and pathological aging in mice and humans separately. Although the proteomic signatures were only moderately correlated, this analysis highlighted a subset of proteins that were consistently affected in mice and humans (supplemental Fig. S3, *E* and *F* and supplemental Table S10). To investigate the functional relevance of our findings, we focused on matrisome that changed consistently during chronological aging in both mice and humans, as well as in decellularized mouse tissue. These included immunoglobulin heavy constant mu (IGHM), lactadherin (MFGE8), vitronectin (VTN), and collagen VI α6 (COL6A6), which were increased, and fibulin-5 (FBLN5) which was decreased in old heart tissue (Fig. 5*A*). All significantly affected proteins (*p* < 0.05 in all conditions) are shown in supplementary data (supplemental Fig. S5*A*). We then evaluated whether the mRNA transcript levels of three proteins, *Mfge8*, *Col6a6*, and *Vtn*, were also altered by aging. Surprisingly, no age-dependent increase in transcription was observed (Fig. 5*B*), suggesting that the increased accumulation of all three proteins in the aged LV may be due to an earlier increased transcription (as it was shown in the aorta during ageing (28), or alternatively, an impaired remodelling of the matrisome. We decided to focus our attention on lactadherin (MFGE8) in cardiac aging because: (i) it showed age-dependent changes in its abundance in mice and humans (Fig. 2*F*), (ii) it is among the most affected ECM proteins (Fig. 5*A* and supplemental Fig. S2*H*), it showed the highest fold change (FC) in DT (enrichment of ECM) and (iii) there is little known about its role in cardiac aging, whereas the role of other ECM proteins such as collagen VI α6 (14) and vitronectin (29) in cardiac aging has already been explored. We then analyzed single-cell transcriptome data to identify the major cell type expressing *Mfge8* (30). We found *Mfge8* mRNA to be broadly expressed across heart cells and it displayed the highest median levels in myofibroblasts, smooth muscle, endocardial, and endothelial cells (Fig. 5*C*).

The age-dependent increase of lactadherin in mice was confirmed by immunofluorescence (Fig. 5*D*). A high percentage of lactadherin accumulated in the large vessels of old mice (Fig. 5*E* and supplemental Fig. S4*B*), particularly in the extracellular space between endothelial and smooth muscle cells (Fig. 5*F*). It is also interesting to note that the accumulation of lactadherin in the extracellular space of SMCs decreased during aging. In addition, we confirmed that *Mfge8* was strongly expressed in coronary artery endothelial cells and in endocardial cells with aging by analysing the Tabula muris senis single-cell RNAseq dataset (31) (supplemental Fig. S4*C*).

Overall, we found that lactadherin is significantly altered in the matrisome during cardiac aging. This protein increased with age in native and decellularized cardiac tissue from old mice and old humans. Its age-related change was not accompanied by corresponding gene expression changes. Accumulation of lactadherin was observed in large blood vessels at the interface between endothelial cells and smooth muscle cells.

### Short- and Long-Term Stimulation with Lactadherin Induces Activation of Age-related Kinases and Signalling Pathways

**Fig. 6:**
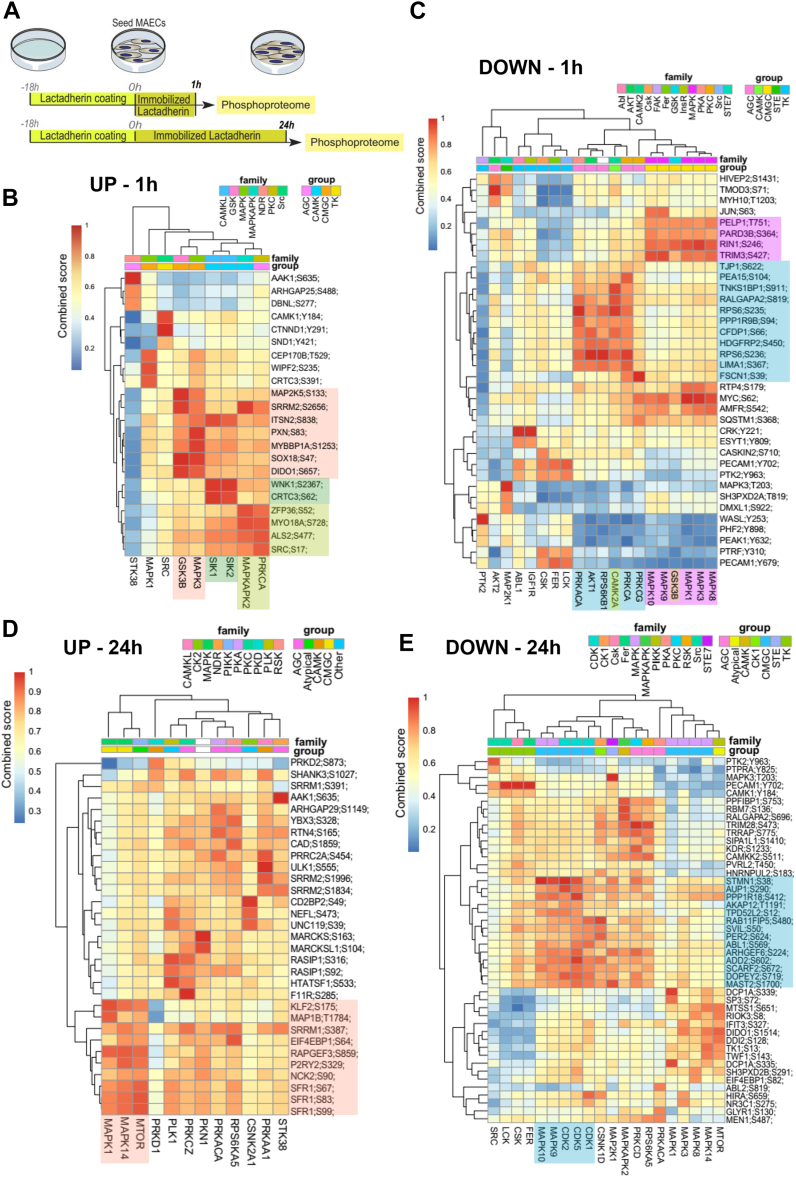
**Phosphoproteomic analysis of MAECs cultured at different time points on a coating of lactadherin/immobilized lactadherin.***A*, scheme illustrating the experimental setup: 10 μg/ml lactadherin was used to coat the plate. A clustered heatmap highlighting the (*B*) up- and (*C*) downregulated phosphosites and predicted kinases regulated by immobilized lactadherin in MAECs after 1 h. A clustered heatmap highlighting *D* up and *E* down regulated phosphosites and predicted kinases regulated by immobilized lactadherin in MAECs at 24 h. The score from 0 (*dark blue*) to 1 (*red*) shows the combined kinase-substrate score determined by the PhosR method (32) for the affected phosphosites (*each row*). A higher combined score (*red*) indicates a better match with a kinase motif (*column*) and the kinase-substrate phosphorylation profile of a phosphosites (*row*). For example, in (*B*), phosphosite S133 of MAP2K5 (*row*) can be strongly phosphorylated by GSK3B (*column*). A high combined score of GSK3B and MAPK3 (belonging to the same kinase group, the CMGC group (*orange*)) in association with a cluster of several upregulated phosphosites clearly indicates that these kinases can regulate the cellular processes induced by lactadherin at 1 h.

To mechanistically investigate the effects of lactadherin on cell aging, the recombinant protein was immobilized in tissue culture dishes, followed by culture of mouse aortic endothelial cells (MAECs) on the coating for one or 24 h, and investigation of the regulation of signaling pathways by phosphoproteomic analysis (Fig. 6*A* and supplemental Fig. S5*A*). The reproducibility of phosphorylation changes is evidenced by the correlation heatmaps, showing the clustering of experimental groups (supplemental Fig. S5, *B* and *C*). We used PhosR (32) to derive global kinase-substrate relationships for the quantified phosphosites, and we found that the phosphoproteome of MAECs at 1 h (Fig. 6, *B* and *C*) was strongly different from that at 24 h (Fig. 6, *D* and *E*). The kinase dendrograms showing the up-regulated (Fig. 6*B* and supplemental Table S11) and down-regulated (Fig. 6*C* and supplemental Table S12) phosphosites at 1 h suggest that three major kinase groups are responsible for the changes in the phosphoproteome of MAECs. CAMK kinases (*e.g.*, MAPKAPK2 and SIK isoforms) and CMGC kinases (GSK3B and MAPK3) showed a higher kinase substrate score for upregulated phosphosites (Fig. 6*B*), whereas AGC kinases (*e.g.*, PRK isoforms, CAMK, RPS6KB1 and AKT1) and CMGC kinases (*e.g.*, GSK3B and several MAPK isoforms) showed a higher score for downregulated phosphosites (Fig. 6*C*). Up-regulated phosphosites of GSK3B and MAPK3 may lead to activation of age-related signaling pathways (33, 34), whereas down-regulation of AKT1-related phosphosites may indicate down-regulation of pro-survival signaling pathways.

At 24 h, the affected phosphoproteome of MAECs mainly comprised phosphosites regulated by AGC, atypical, and CMGC kinase groups. CMGC kinases (MAPK14 and MAPK1) and MTOR showed a high kinase-substrate score for several up-regulated phosphosites (Fig. 6*D* and supplemental Table S13). Importantly, MTOR kinase has been shown to be an important trigger for the senescence-associated secretory phenotype (SASP) (35). The group of CMGC kinases (*e.g.*, CDK isoforms, MAPK9, and MAPK10) showed a high score for down-regulated phosphosites (Fig. 6*E* and supplemental Table S14). CDK kinases, such as CDK1, CDK2, and CDK5, are involved in cell cycle control and cellular senescence (36).

**Fig. 7:**
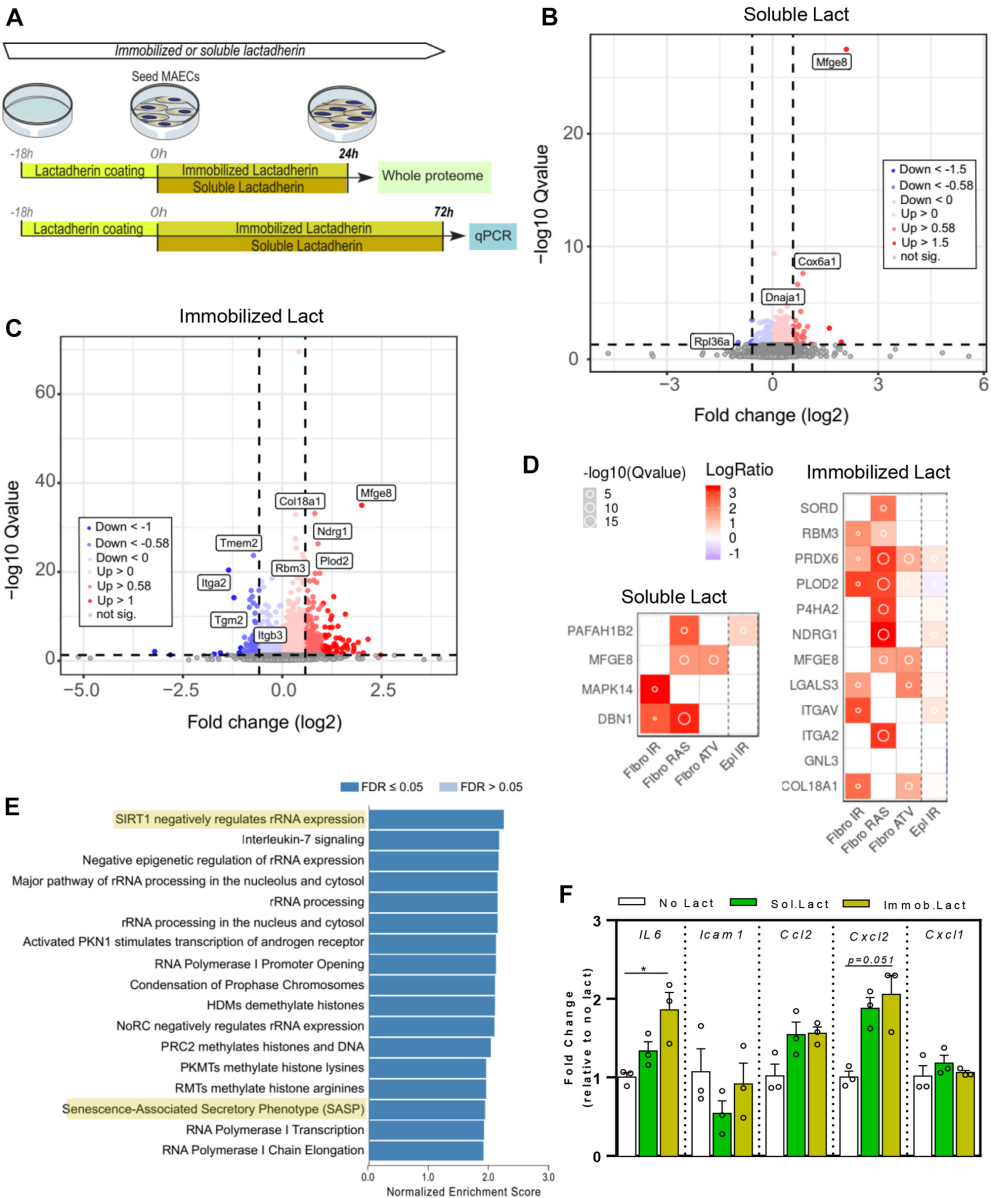
**Lactadherin affects the whole proteome and transcriptome of MAECs.***A*, schematic representation of the experimental setup; 10 μg/ml or 500 ng/ml lactadherin was used for immobilized and soluble stimuli, respectively. Volcano plots highlight the *up* (*red*) and *down* (*blue*) significantly changed proteins (q-value <0.05 and absolute log2 fold change >0.58) after treatment with *B*, soluble lactadherin (Lact) and *C*, immobilized Lact for 24 h. Proteins not affected are shown in *grey*. Figure 7, *B* and *C*: The presence of lactaderin/Mfge8 likely indicates contamination by the recombinant protein used in the experimental procedure. *D*, heatmaps showing the proteins affected by soluble and immobilized lactadherin generated using SASP Atlas, http://www.saspatlas.com (38). The top 20 significantly affected proteins (ranked on q value) were listed in the SASP query tool, which displays a heatmap depicting the presence and secretion levels in different SASPs; human fibroblasts after induction of senescence by X-ray irradiation (Fibro IR), RAS overexpression (Fibro RAS) and Atazanivir treatment (Fibro ATV); human renal epithelial cells after induction of senescence by X-ray irradiation (Epi IR). *E*, GSEA shows the most affected pathways in MAECs cultured on immobilized lactadherin for 24 h using WebGestalt (64). *F*, bar plot shows relative gene expression quantified by RT-qPCR for different genes in MAECs. Data are shown as average ± SEM of fold change, normalized to control (No Lact) average transcript levels. n = 3; ∗∗*p* < 0.01; ∗*p* < 0.5, ordinary two-way ANOVA.

Next, the whole proteome of MAECs cultured for 24 h with lactadherin was evaluated by MS-based proteomics. MAECs were cultured in the presence of immobilized lactadherin (lactadherin-coated plate) or with soluble protein (recombinant mouse lactadherin-enriched medium) (Fig. 7*A*). This is due to the fact that part of the lactadherin secreted into the extracellular space is soluble while the remaining is sequestered in the ECM (37). The volcano plots show that both soluble (Fig. 7*B*) and immobilized (Fig. 7*C*) lactadherin induced proteomic remodelling in MAECs. However, this was stronger when immobilized lactadherin (Fig. 7*C*) was used, as it had a greater impact on the overall proteome of MAECs compared to the soluble protein (Fig. 7*B*). In MAECs stimulated with immobilized lactadherin, we found 387 significantly altered proteins (q-val <0.05 & Avg.Ratio (log2) >0.58), whereas soluble lactadherin caused only minor changes in the proteome (supplemental Fig. S5*D*). Of these proteins, the 20 most altered proteins, corresponding to the lowest q-value, for soluble and immobilized lactadherin (supplemental Fig. S5*E*) were further analysed (supplemental Tables S15 and S16). Soluble lactadherin resulted in an increase in FAM118A protein, glucosamine-6-phosphate isomerase 1 (Gnpda1), multiple coagulation factor deficiency 2 (Mcfd2) protein, cytochrome c oxidase subunit 6A1 (Cox6a1), and others, which showed the highest fold changes (Fig. 7*B* and supplemental Fig. S5*E*). The immobilized lactadherin promoted a large number of significantly altered proteins, such as an increase in prolyl 4-hydroxylase subunit alpha-2 (P4ha2), procollagen-lysine 2-oxoglutarate 5-dioxygenase 2 (Plod2), protein NDRG1 (Ndrg1), and many others (Fig. 7*C* and supplemental Fig. S5*E*). We then investigated whether the top 20 altered proteins (supplemental S5*E*) were associated with SASP proteins observed in other types of induced senescent cells, as shown in the SASP Proteome Atlas (38). Heatmaps showed that MAECs stimulated with immobilized lactadherin had a large number of altered proteins (12 of 20 proteins) belonging to the SASP proteome atlas (Fig. 7*D*). This suggests that immobilized lactadherin may trigger a senescence program in MAECs. Because immobilized lactadherin had a greater impact on the whole proteome of MAECs, we applied GSEA to identify the signaling pathways significantly altered by immobilized lactadherin. We observed an increase in several signaling pathways, some of which are related to aging, such as SASP and SIRT1, which negatively regulates rRNA expression, which is also related to cellular energy levels (39) (Fig. 7*E*).

To investigate the effects of lactadherin at the functional level, we cultured MAECs for 3 days with immobilized (Immob) or soluble (Sol) recombinant lactadherin and monitored the expression of various pro-inflammatory components by qRT-PCR (Fig. 7*A*). Our results showed that cells cultured on immobilized lactadherin had increased expression of pro-inflammatory *IL6* and *Cxcl2*, although not statistically significant in the latter case (Fig. 7*F*).

Taken together, our proteomic data show that MAECs cultured on immobilized lactadherin for up to 24 h activate age-related signaling pathways, *i.e.*, increase GSK3B, MAPK, and MTOR activity and decrease CDK activity, and secrete proteins related to SASP and inflammation.

## Discussion

Our study provides new insights into proteomic remodeling of left ventricular myocardial tissue during chronological and pathological (HGPS) aging. Here, we report that matrisome was the most affected cellular compartment during chronological ageing of the LV, whereas nuclear and mitochondrial proteins were more affected during pathological accelerated aging (HGPS). Integration of our proteomic data shows that lactadherin is a cardiovascular aging marker that is upregulated in mice and humans and accumulates in the vasculature of LV myocardium. We also show that immobilized lactadherin activates age-related kinases and signaling pathways in mouse aortic endothelial cells.

The current study focuses on ECM and factors that are immobilized by the ECM. To show the effect of lactadherin, we have used both soluble and immobilized molecules since it is not clear whether they act in the same way. As control, cells were cultured in tissue culture polystyrene.

Chronological aging significantly impairs LV matrisome remodeling. Proteomic remodeling of LV during aging has been described in mice and humans (11, 12, 40); however, few studies have highlighted age-related changes in ECM and ECM-associated proteins (14, 40). In the current study, we found that the ECM and ECM-associated proteins were most affected by chronological aging. We used two different strategies to detect changes in the matrisome during aging: (i) whole LV lysate and (ii) decellularized LV tissue. We decellularized LV to enrich the ECM, which allowed us to quantify 204 ECM and ECM-associated proteins, about half of which were significantly affected by ageing, in contrast to 44 ECM proteins previously identified (14). A potential limitation of our study is related to the fact that an insoluble ECM fraction was observed during the processing of the samples (both LV lysate as well as in decellularized LV tissue). This procedure left some fraction of ECM in the pellet that was not characterized. Decellularization allowed us to detect several age-related changes in matrisome that were not identified in the whole LV lysate, such as an increase in laminin subunit alpha-2 (lama2) and stromal cell-derived factor 1 (sdf-1). Our proteomic results obtained in decellularized LV tissue also highlighted important differences from other results reported in other tissues. For example, in the current study, we demonstrated a decrease in SMOC-2 protein in aged LV, whereas the same protein is increased in aged skeletal muscle (10). Thus, ageing could differentially affect the matrisome composition of different tissues.

Combining proteomic data from chronological aging in mice and humans showed that aging resulted in upregulation of matrisome such as immunoglobulin heavy constant mu, lactadherin/MFGE8, collagen VI α6, vitronectin, and others, while fibulin-5 was downregulated. Because transcript levels remained unchanged for some of the proteins studied, it is possible that the mechanisms involved are independent of transcriptional changes. Indeed, altered protein turnover may explain the accumulation of proteins in the ECM of the aged heart. Several studies have described an uncoupling between proteome and transcriptome during aging (41, 42). Therefore, it is possible that the observed accumulation of some ECM proteins is triggered, at least in part, by posttranscriptional mechanisms that regulate protein abundance, including altered protein degradation by matrix metalloproteinases (MMPs), elastases and tissue inhibitors of metalloproteinases (TIMPs), as observed in several aged tissues such as skin, aorta, and heart (43, 44, 45).

Here, we also investigated the proteomic remodeling of the heart during pathological aging (HGPS) in mice and humans and found a different proteomic profile compared with chronological aging. Although a recent study documented the proteomics of hearts from progeroid and chronologically aged mice, the study was limited to highlighting the main differences at the matrisome level between the progeroid and physiologically aged mouse heart and did not perform the same type of characterization in human tissues (13).

Lactadherin, a marker of cardiac aging, is a glycoprotein originally found in milk and mammary epithelial cells (28). This protein has been shown to have multiple biological functions in various tissues. For example, lactadherin acts as an antiviral protein in milk, facilitates fertilization of oocytes, mediates the binding of macrophages to apoptotic cells, and modulates blood vessel growth (37, 46). Lactadherin has also been linked as a marker of vascular aging, particularly in smooth muscle cells (28, 47, 48). For example, it has been shown that lactadherin accumulates in blood vessels during aging (28). In non-human primates and humans, lactadherin levels in the aorta increased, approximately nine and 6.5-fold, respectively, during aging. Angiopoietin II was shown to induce lactadherin expression in vascular smooth muscle cells (VSMCs) isolated from rat aorta (28). Lactadherin also promotes ERK1/2 phosphorylation, induces proliferation of VSMCs (49) and migration (28), regulated by Arp2-mediated actin polymerization (50), and may contribute to the thickening of the intima–media of the aged arterial wall. Recently, it has been shown that lactadherin promotes a proinflammatory phenotype in aged VSMCs and is required for age-related proinflammatory aortic remodeling (51, 52). However, the effect of lactadherin in endothelial cells during aging is poorly understood, particularly in the context of the heart. Our results show that lactadherin accumulates at the interface between endothelial cells and smooth muscle cells, particularly in large, aged vessels. Yet, it remains to be determined the effect of lactadherin in cardiac non-vascular cells (*e.g.* myofibroblasts, endocardial cells, etc) and this should be investigated in the near future. It is possible that the effect of lactadherin in these cells is mainly mediated by soluble lactadherin and not immobilized lactadherin.

The rapid increase in the elderly population, which is inherently at increased risk for CVDs, requires more specific biomarkers of cardiac ageing to better stratify risk and guide therapy. Therefore, lactadherin could be an important marker for stratifying patients. Among established biomarkers available to predict cardiovascular disease risk (53), N-terminal pro-B-type natriuretic peptide has been associated with cardiac dysfunction due to age-related remodeling of the myocardium (54). Moreover, serum levels of lactadherin increase with aging and have been proposed to serve as a potential marker of atherosclerosis severity in humans (55). Telomere shortening has also been linked to loss of function of the cardiovascular system and has been proposed as a cardiovascular aging marker (56, 57).

Lactadherin contributes to active age-related kinases and signaling pathways. We have shown that the phosphoproteome of MAECs induced by immobilized lactadherin was regulated in a timely manner by MAPK3 and GSK3B, MAPK1, MAPK14, and MTOR, as well as by CDKs. Chronic inflammation increases with age, and GSK3 has been shown to regulate the inflammatory response by promoting the stability and nuclear localization of NFkB, leading to the production of pro-inflammatory cytokines (33). More recently, the role of MAPKs in slowing cell growth, resistance to apoptosis, and other behaviors of senescent cells have also been demonstrated (58, 59, 60). In particular, ERK1/2 (MAPK1/3) and p38 (MAPK14) are most closely associated with cellular senescence (60). Aging and the pathophysiology of aging are also modulated by the MTOR pathway, the inhibition of which improves the lifespan of several organisms (61). These signaling pathways may also modulate the SASP phenotype in response to senescence-inducing stimuli, such as p38, which enhances DNA damage and promotes transcription of SASP genes such as IL6, IL8, and GM-CSF (62).

In summary, we provide extensive proteomic data describing the changes in mice and humans LV during chronological aging and accelerated aging disease (HGPS/pathological aging). Importantly, we have identified significant changes in matrisome during chronological aging that were examined in detail using a decellularization approach. Matrisome remodeling appears to depend on posttranscriptional mechanisms that regulate protein levels. We identified lactadherin, an ECM protein that was simultaneously upregulated during chronological and pathological aging in mice and humans. Its identification was strengthened by analysis of the matrisome enriched by decellularization. In endothelial cells, increased levels of lactadherin, as observed during aging, triggered an important age-related phenotype, attributable to the activation of various kinases and a pro-inflammatory response.

## Data Availability

The mass spectrometry proteomics data underlying this article are available in ProteomeXchange Consortium *via* the PRIDE (63) partner repository, and can be accessed with the dataset identifier PXD039548 and PXD040234. The annotated spectra can be browsed from the ".sne" files using the freely available Spectronaut Viewer https://biognosys.com/software/viewers/.

The codes supporting the study are available from the corresponding authors upon request.

## Supplemental data

This article contains supplemental data (19, 20, 32, 65, 66, 67, 68, 69, 70, 71, 72).

## Conflict of interest

The authors declare that they have no known competing financial interests or personal relationships that could have appeared to influence the work reported in this paper.
