# Supplementary material for: Remodeling of the Cardiac Extracellular Matrix Proteome During Chronological and Pathological Aging
Source: Mol Cell Proteomics. 2023 Dec 21;23(1):100706. doi: 10.1016/j.mcpro.2023.100706 (PMC10828820; doi:10.1016/j.mcpro.2023.100706)
Supplement: Supplementary Figures [file mmc16.docx]

**Remodelling of the cardiac extracellular matrix proteome during chronological and pathological aging**

**Authors:** Deolinda Santinha^1,2^, Andreia Vilaça^2,3^, Luís Estronca^1,2^, Svenja C. Schüler^4^, Catherine Bartoli^5^, Maximillian Quaas^6^, Annachiara De Sandre-Giovannoli^5,7^, Tilo Pompe^6^, Arnaldo Figueiredo^8^, Alessandro Ori^4^* and Lino Ferreira^1,2^*

**Supplementary Information:**

**1. Methods**

**Left ventricle dissection**

Left ventricle (LV) wall was isolated from mice after sacrificing them by using 0.5 ml/min CO_2_ inhalation for 5 min. For subsequent mass spectrometry or decellularization procedure, the LV wall was cut into similar portions and were snap frozen in liquid nitrogen in 1.5 ml tubes (Eppendorf, 0300120.086) and stored at -80°C. For histological analyses, LV tissues were isolated from mice after sacrificing them by using 0.5 ml/min CO_2_ inhalation for 5 min. They were cut in half, snap-frozen using liquid nitrogen in freezing medium in an aluminum foil and stored at -80 °C.

Freezing medium (1 part 30% sucrose (w/v, 21938, Affymetrix) and 2 parts Thermo Scientific™ RichardAllan Scientific™ Neg-50™ Frozen Section Medium (6502, Thermo Fisher))

**Decellularization of the mouse left ventricle (LV)**

Similar LV portions (6-9 mg) from 5 young and 5 old animals were decellularized following the protocol adapted from Silva et al. 2016 ^1^. Briefly, LV tissues were incubated for 18 h in hypotonic buffer (10 mM Tris HCl/0.1% EDTA, pH 7.8 (Alfa Aesar, A10713) and washed three times in PBS (1 h per wash) before decellularization with 0.2 % sodium dodecyl sulphate (SDS) (VWR, 444464T) in 10 mM Tris. HCl (Merck, 10812846001) pH 7.8 for 24 h. Then, the decellularized tissues were washed three times (20 min per wash) with the hypotonic wash buffer (10 mM Tris HCl, pH 7.8) and incubated in DNAse solution (50 U/mL DNAse (Qiagen, 79254)/10 mM Tris HCl, pH 7.8) for 3 h at 37 ºC. To remove residual detergent and DNAse, multiple washes (20 min each) were performed before evaluating the evaluating the efficacy of decellularization by biochemical, histochemical, and immunohistochemical analyses. With the exception of the DNAse treatment, all steps were performed under agitation at 165 rpm at 25ºC, and all solutions were supplemented with a protease inhibitor cocktail (Roche, 11836170001). The decellularized LV tissue portions were stored at -80 ºC until further use. For a schematic representation of the decellularization protocol, see Figure 3a.

**Biochemical quantification of dsDNA, total proteins, collagens and sGAGs in native and decellularized LV tissue**

Double-stranded deoxyribonucleic acid (dsDNA) and major ECM components were quantified from decellularized (DT) and compared with native (NT) LV mouse heart tissue. NT and DT samples were digested in 500 µl papain digestion solution (3.88 units/ml Papain, 100 mM L-cysteine (Sigma-Aldrich, 30089), 100 mM Sodium phosphate (Sigma-Aldrich, 71496), 5 mM EDTA (Alfa Aesar, A10713), pH 6.5) for 20 h at 60˚C with agitation. The dsDNA content was determined using the DNA Quantitation Kit (Sigma-Aldrich, DNAQF) according to the manufacturer’s instructions. Fluorescence intensity was measured using the Synergy H1 microplate reader (BioTeck, Aligent). The quantity of protein, collagen (soluble, insoluble, and total), and sulphated glycosaminoglycans (sGAG) was quantified using BCA assay (Thermo Scientific, PI23225), QuickZyme Total Collagen assay (Quickzyme Biosciences, QZBTOTCOL1), and Glycosaminoglycan assay Blyscan (Biocolor, B1000), respectively, according to the manufacturer’s instructions. Absorbance of the sample was measured using the Synergy H1 microplate reader. Data were expressed as measured component mass normalized to tissue wet weight or protein content.

**Histochemical analysis of native and decellularized mouse LV tissue**

OCT-embedded NT and DT were sectioned in a cryostat (Leica Biosystems), and the cryosections were fixed for 5 minutes at room temperature (RT) with 10 % neutral buffered formalin solution (Sigma, HT501128) and washed three times in PBS. Staining with the Picrosirius Red Stain Kit (Polysciences, 24901) was performed according to the manufacturer’s instructions. After most water was removed, samples were rinsed in Xylene (Sigma, 534056) and mounted in Xylene-containing mounting medium (Leica, 14046430011). Microscopic images were acquired with an Axio Scan.Z1 system (Carl Zeiss) using a Plan Apochromat 20x/0.8 objective. Bright field images were taken with transmitted light (LED) and a 3 CCD Hitachi colour camera. Fluorescence images were acquired using a Colibri 7 for excitation (385nm, 475nm and 555nm) and an Orca Flash 4.0 V3 monochromatic CMOS camera.

**Immunohistochemical analysis of native and decellularized mouse LV cryosections**

Cryosections of native and decellularized left ventricular heart tissue (6μm thick) were fixed with cold acetone (Fisher Chemical, A/0600/21) for 5 min at RT, washed twice with PBS, and then permeabilized for 5 min at RT (0.1 % Triton X-100 (Sigma-Aldrich, X100). Washing with PBS was repeated three times, and cryosections were incubated with a blocking solution of 5 % of Bovine Serum Albumin (BSA) (Sigma-Aldrich, A4503) in PBS for 1 h at RT. Sections were incubated overnight at 4 °C with primary antibodies in 1 % BSA against the following proteins: α-Actinin (Sarcomeric) (Sigma-Aldrich, A7811, mouse clone EA-53, 1:100) and Collagen I (Abcam, ab34710, rabbit IgG, 1:100). After washing three times with PBS, the sections were incubated for 1 hour at RT with the respective secondary antibody: Donkey anti-mouse IgG (Alexa Fluor&reg 488, Invitrogen, A21202) and Goat anti-rabbit IgG (Alexa Fluor 633, Invitrogen, A-21070) 1:800 in 1 % BSA in PBS. After washing, nuclei were stained with DAPI (2 μg/mL in PBS) (Sigma, D9542) at RT for 5 min followed by three washing steps using PBS and mounting in VECTASHIELD® HardSet Antifade Mounting Medium H-1400 (Vector Laboratories, H-1400). Stained cryosections were observed with high-content fluorescence microscope (IN Cell 2200, GE Healthcare) or using an LSM 710 point-scan confocal laser microscope (Zeiss) with a 40× oil-immersion objective.

**Scanning Electron Microscopy of decellularized mouse LV tissue**

Decellularized LV portions were fixed in 2.5% Glutaraldehyde (Sigma-Aldrich, 49630) in 10% neutral buffered formalin Buffer overnight at 4°C. Samples were placed on self-adhesive carbon substrates (12 mm, Agar Scientific) and visualized using a variable-pressure scanning electron microscope (FlexSEM 1000, Hitachi) at an accelerating voltage of 10 kV. SEM images were visualized using the open-source Image J software (Rasband, W.S., ImageJ, U. S. National Institutes of Health, https://imagej.nih.gov/ij/, 1997-2019).

**Atomic Force Microscopy of native and decellularized mouse LV tissue**

Atomic Force Microscopy (AFM) analyses were performed using a Nanowizard 3 AFM (JPK Instruments) mounted on an inverted optical microscope (Observer.D1, Zeiss). A tipless, 300 μm cantilever with a nominal spring constant of 0.06 N/m (MikroMasch® SPM probes) was used for elasticity and height measurements. For elasticity measurements, colloidal force probes were prepared by attaching glass beads (10 μm diameter) to the apex of a tipless cantilever. The Young’s modulus was extracted from the force-displacement curves of the samples using AFM data processing software (JPK Instruments).

**Lysis of the native and decellularized LV tissues for MS analysis**

Native and decellularized left ventricular tissues were cut into fragments in Precellys Tubes Keramik-Kit 1,4/2,8 mm tube (VWR, 431-0170), 400 μl of PBS was added, and homogenized in a Precellys 24 homogenizer (Bertin) for 30 seconds at 4 ºC. An additional volume of 200 μl PBS was added to all samples to homogenize a second time for 30 seconds. The homogenates were aliquoted before subsequent lysis (NT 20 μl; DT 20 μl). For lysis, samples were filled up to 100 μl with PBS and 20μl of 10 % (w/v) sodium deoxycholate (Sigma, 30970) was added and then boiled at 95°C for 10 min. Samples were allowed to cool for 10 min at RT before adding 80 μl of 10 M Urea in 250 mM ammonium bicarbonate (Roth, T871.2). Samples were sonicated using a Bioruptor Plus (Diagenode) for 10x 1 min on, 30 sec off at 20 °C using the highest settings, followed by a spun down at 21,000x *g* for 1 min and transferred to a new tube. SDS-PAGE and BCA assay analyses were performed to assess the protein concentration of each sample.

**Sample preparation for MS analysis**

Tissue lysates (50μg) were reduced by 10 mM dithiotreitol (DTT) (Roth, 6908.3) at 37 °C for 30 minutes and then alkylated with 20 mM iodoacetamide (Sigma, I1149) for 30 minutes at RT in the dark. Proteins were precipitated with 100 % Trichloroacetic Acid (TCA) (Sigma, T6399) at a volume ratio of 1:4for 30 min on ice, centrifuged at 21,000xg for 20 min at 4°C, and the pellet was washed twice with 1 ml of pre-cooled 10% TCA followed by two washes with 100% ice-cold acetone (Biosolve, 0001037801BS). The pellets were air-dried before adding the appropriate volume of digestion buffer (3 M Urea (Sigma, U6504), 100 mM HEPES (Sigma, H3375), pH 8) to achieve a protein concentration of 1 – 3 mg/ml. Protein digestion was started by adding LysC (Wako Chemical GmbH, 125- 05061) at a ratio of 1:100 (w/w) enzyme:protein for 4h at 37 °C with shaking (1000 rpm for 1h, then 650 rpm). Samples were then diluted 1:1 with MilliQ water and trypsin (Promega, V5111) was added at a 1:100 (w/w) enzyme:protein ratio to complete digestion overnight at 37 °C with shaking (650 rpm). Digested samples were acidified with Trifluoroacetic acid (TFA) (Biosolve, 0020234131BS) to a final concentration of 2% (v/v) and then desalted using Sep Pak C18 cartridges (Waters, WAT054945) according to the manufacturer’s instructions (Waters Corporation, Milford, MA, USA). Eluates were dried in a speed vacuum centrifuge and then dissolved at a concentration of 1 µg/µL in reconstitution buffer (5% (v/v) acetonitrile (Biosolve, 0001204102BS), 0.1% (v/v) formic acid (Roth, 4724.3) in Milli-Q water. The reconstituted peptides were used for TMT labelling.

**Protein solubilisation of FFPE mouse and human samples**

Tissue sections were deparaffinised in xylene for 2 × 5 minutes, rehydrated in 100% ethanol for 2 × 5 minutes, and then washed in 96% (v/v), 70% (v/v), 50% (v/v) ethanol and MilliQ water for 1 × 5 min each. Sections were gently scraped using a scalpel and collected into PCR tubes containing 100 μL protein solubilisation buffer (80 μM Tris pH 8.0, 80 μM DTT, and 4% SDS) and processed directly. Samples were sonicated with a bioruptor for 25.2 minutes (15 cycles: 1 min on, 30 sec off) at 20°C, and then boiled at 99°C for 1h. These last two steps were performed twice. After protein solubilisation, the cysteine residues were alkylated by adding 200 mM iodoacetamide to a final concentration of 15 mM (incubation for 30 min at room temperature in the dark) and 10 μL of 200 mM DTT was added to quench the reaction. These proteins were then precipitated with four volumes of ice-cold acetone to one volume of sample and allowed to stand overnight at − 20 °C. Samples were then centrifuged at 20,800×g for 30 min at 4 °C. After removal of the supernatant, precipitates were washed twice with 500 μL 80% (v/v) acetone (ice cold). After each wash step, samples were vortexed and then centrifuged again for 2 min at 4 °C. Pellets were further digested, acidified, and desalted as described in Sample Preparation for MS Analysis**.**

**TMT labelling**

The resuspended peptides were buffered to pH 8.5 with 1 M HEPES before labelling. For the experiment, 20 μg of peptides were used for each labelling reaction. TMT-10plex reagents (Thermo Fisher, 90111) were reconstituted in 41 µL acetonitrile (Biosolve, 0001204102BS). TMT labelling was performed in two steps by adding 2x of the TMT reagent per µg of peptide (e.g, 40 µg TMT reagent for 20 µg peptides). The first amount of TMT reagent was added to the samples at RT, followed by incubation in a thermomixer (Eppendorf) for 30 min under constant shaking at 600 rpm. After incubation, a second portion of TMT reagent was added and incubated for an additional 30 minutes. Next, the efficiency of the labelling was checked by MS. Then, samples were pooled equally (200 μg total), desalted using two wells of Waters Oasis® HLB µElution Plate 30 µm (Waters, 186001828BA), and subjected to high pH fractionation prior to MS analysis. Samples were labelled according to the following table.

| **Mouse** | Young | | | | | Old | | | | |
| --- | --- | --- | --- | --- | --- | --- | --- | --- | --- | --- |
|  | 1 | 2 | 3 | 4 | 5 | 1 | 2 | 3 | 4 | 5 |
| **Native Tissue** | 126 | 127N | 127C | 128N | 128C | 129N | 129C | 130N | 130C | 131 |
| **Decell Tissue** | 129N | 129C | 130N | 130C | 131 | 126 | 127N | 127C | 128N | 128C |
|  | | | | | | | | | | |
| **Human** | Young | | | | Old | | | | Progeria | |
|  | 1 | 2 | 3 | 4 | 1 | 2 | 3 | 4 | 1 | 2 |
|  | 126 | 127N | 127C | 128N | 128C | 129N | 129C | 130N | 130C | 131 |
|  | | | | | | | | | | |
| Mouse **Progeria** | Wild-type | | | | | Progeria | | | | |
|  | 1 | 2 | 3 | 4 | 5 | 1 | 2 | 3 | 4 | 5 |
|  | 126 | 127N | 127C | 128N | 128C | 129N | 129C | 130N | 130C | 131 |

**High pH peptide fractionation**

Off-line reversed-phase high pH fractionation was performed using an Agilent 1260 Infinity HPLC system equipped with a binary pump, degasser, variable wavelength UV detector (set at 220 and 254 nm), Peltier-cooled auto sampler (set at 10 °C), and fraction collector. The column used was a Waters XBridge C18 column (3.5 µm, 100 x 1.0 mm, Waters) with a Gemini C18, 4 x 2.0 mm SecurityGuard (Phenomenex) cartridge as a guard column. The solvent system consisted of 20 mM ammonium formate (20 mM formic acid (Biosolve, 00069141A8BS), 20 mM (Fluka, 9857) pH 10.0) as mobile phase (A) and 100% acetonitrile (Biosolve, 0001204102BS) as mobile phase (B). Separation was performed at a mobile phase flow rate of 0.1 mL/min using a non-linear gradient of 95% A to 40% B for 91 minutes. Forty-eight fractions were collected along with the LC separation, which were subsequently pooled into 23 or 24 fractions. The pooled fractions were dried in a speed vacuum centrifuge and then stored at -80 °C until MS analysis.

**Data acquisition from TMT-labelled samples**

For TMT experiments, fractions were resuspended in 20 µL reconstitution buffer (5 % (v/v) acetonitrile (Biosolve, 0001204102BS), 0.1 % (v/v) TFA in water), and 5 µL were injected into the mass spectrometer. Peptides were separated using the nanoAcquity UPLC system (Waters) fitted with a trapping (nanoAcquity Symmetry C18, 5 µm, 180 µm x 20 mm) and an analytical column (nanoAcquity BEH C18, 2.5 µm, 75 µm x 250 mm). The outlet of the analytical column was coupled directly to an Orbitrap Fusion Lumos (Thermo Fisher Scientific) using the Proxeon nanospray source. Solvent A was water containing 0.1% (v/v) formic acid and solvent B was acetonitrile, 0.1 % (v/v) formic acid. Samples were loaded with a constant flow of solvent A at 5 µl/min, onto the trapping column. The trapping time was 6 minutes. Peptides were eluted over the analytical column at a constant flow rate of 0.3 µl/ min at 40 °C. During the elution step, the percentage of solvent B increased linearly from 5 % to 7 % in the first 10 min, then from 7 % B to 30 % B in the following 105 min and to 45 % B by 130 min. Peptides were introduced into the mass spectrometer via a Pico-Tip Emitter 360 µm OD x 20 µm ID; 10 µm tip (New Objective) and a spray voltage of 2.2kV was applied. The capillary temperature was set at 300 °C. Full scan MS spectra with a mass range of 375-1500 m/z were acquired in profile mode in the Orbitrap with a resolution of 60000 FWHM using the quad isolation. The RF on the ion funnel was set to 40 %. The filling time was set to a maximum of 100 ms with an AGC target of 4 x 105 ions and 1 microscan. The peptide monoisotopic precursor selection was enabled along with relaxed restrictions if too few precursors were found. The most intense ions (instrument operated for a 3 second cycle time) from the full scan MS were selected for MS2, using quadrupole isolation and a window of 1 Da. HCD was performed with a collision energy of 35 %. A maximum fill time of 50 ms was set for each precursor ion. MS2 data were acquired with a fixed first mass of 120 m/z and acquired in the ion trap in Rapid scan mode. The dynamic exclusion list was set with a maximum retention time of 60 seconds and a relative mass window of 10 ppm. For MS3, the precursor mass selection window was set to the range 400-2000 m/z, with an exclusion width of 18 m/z (high) and 5 m/z (low). The most intense fragments from the MS2 experiment were co-isolated (using Synchronus Precursor Selection = 8) and fragmented with HCD (65 %). MS3 spectra were acquired in Orbitrap over the mass range of 100-1000 m/z and resolution was set to 30000 FWHM. The maximum injection time was set to 105 ms and the instrument was set not to inject ions for all available parallelizable time. Xcalibur v4.0 and Tune v2.1 were used for data acquisition and raw data processing.

**RT-qPCR**

Total RNA was extracted from left ventricular tissue of young and old mice and from MAECs using the miRNeasy Micro Kit (Qiagen, 217084) or the RNeasy Plus Micro kit (QIAGEN, 74034), respectively. RNA was then quantified at 260 nm using a NanoDrop 2000 spectrophotometer (NanoDrop Technologies, Inc., USA) and immediately stored at - 80 ºC. First-strand cDNA synthesis was performed using qScript (Quanta bio). Real time quantitative PCR (RT-qPCR) of 5 ng RNA used for cDNA synthesis was performed using NZYSpeedy qPCR Green Master Mix (NZYtech, MB224) according to the manufacturer’s instructions at an annealing temperature of 60°C, and detection was carried out in the CFX Connect Real-Time PCR system (Bio-Rad, U.S.A.). Quantification was performed using the comparative ∆Cq method with Gapdh as the reference gene. The primers used in this work (designed by Sigma) are listed in the Table 1.

*Table 1 - Primer pairs used for the RT-qPCR analysis and the respective sequence.*

| Gene | Primer Forward | Primer Reverse |
| --- | --- | --- |
| Gapdh | GGAGAAACCTGCCAAGTATGAT | GTGGGAGTTGCTGTTGAAGT |
| Mfge8 | GTGGAGACAAGGAGTTTTTG | AAACAGGGTACAGCTTTATG |
| Vtn | TACTTGTTCAAGGGTAGTCAG | ATCAACATTGTCTGGTATGC |
| Col6a6 | GGTGGTCCTTCTATTTTCAG | TTCCAATTGTGAACTGTGTC |
| Il6 | ACCTGTCTATACCACTTCAC | GGCAAATTTCCTGATTATATCCA |
| Icam1 | CAGTCTACAACTTTTCAGCTC | CACACTTCACAGTTACTTGG |
| Ccl2 | CAAGATGATCCCAATGAGTAG | TTGGTGACAAAAACTACACAGC |
| Cxcl1 | AAAGATGCTAAAAGGTGTCC | GTATAGTGTTGTCAGAAGCC |
| Cxcl2 | GGGTTGACTTCAAGAACATC | CCTTGCCTTTGTTCAGTATC |

**Immunofluorescence of young and old LV mouse heart tissue**

Sections (6 μm thick) of FFPE mouse LV tissue were cut and mounted on a glass slide, then deparaffinised in xylene and rehydrated in a graded ethanol series. The mouse and human tissue sections underwent heat-mediated antigen retrieval using citrate buffer 10 mmol, 0.05% Tween 20 (pH=6), in a water bath for 20 minutes at 97 ºC. After cooling for 20 minutes at RT, the sections were washed in PBS for 5 minutes and then permeabilized with 0.5 % Triton X-100 (Sigma-Aldrich, X100) for 5 min at RT. Sections were then washed twice with PBS and blocked with 5 % BSA (Sigma-Aldrich, A4503) in PBS for 1 hour at RT. Then, the tissue sections were incubated overnight with the primary antibody in 1% BSA. Finally, after washing three times with PBS, the sections were incubated with the respective secondary antibody for 1 hour at RT and visualized with a high-content fluorescence microscope or Zeiss LSM 710 confocal microscope using a LD PlanAprochromat 20x/1.0 objective or a 40x objective/ 1.4 numerical aperture oil PlanApochromat immersion lens. The antibodies used were: Anti- Milk Fat Globule (MFG)-E8 antibody (R&D systems, AF2805, Goat Polyclonal IgG, 1:100), Anti- Smooth Muscle Actin (SMA) (Dako, M0851, mouse monoclonal, clone 1A4 1:100), and Anti-CD31 antibody (Abcam, ab28364, rabbit polyclonal IgG, 1:50). The secondary antibodies used were: Cy5.5®, Alexa Fluor 680 (Lumiprobe, 47020), donkey anti-mouse IgG (Alexa Fluor 488, Invitrogen, A-21202), goat anti-rabbit IgG (Alexa Fluor 633, Invitrogen, A-21070), 1:800 in 5 % BSA in PBS. After washing, nuclei were stained with DAPI (2 μg/mL in PBS) (Sigma, D9542) at RT for 5 min followed by three washing steps with PBS and mounting in VECTASHIELD® HardSet Antifade Mounting Medium H-1400 (Vector Laboratories, H-1400). Stained cryosections were observed using a high-content fluorescence microscope (IN Cell 2200, GE Healthcare) or an LSM 710 point-scan confocal laser microscope (Zeiss) with a 40× oil immersion objective.

**Phosphoproteomic analysis**

MAECs were grown in basal medium supplemented with 5% FBS and supplements, according to the supplier’s instructions. Cells were used between passages 4 and 6. Serum-depleted cells (18 h in basal medium containing 0.1% bovine serum albumin (BSA)) were trypsinized and seeded in coated wells containing 10ug/ml of recombinant mouse lactadherin for short (1 h) and long (24 h) stimulation. Cells were scraped into 200 µl PBS on ice, the cell suspension was centrifuged at 300 xg for 10 min, and pellets were stored at -20 °C until further use. Pellets were lysed in RIPA buffer (150 mM Sodium Chloride (Roth, P029.2), 1 % Triton X-100 (v/v, Roth, 3051.3), 0.5 % Sodium Deoxycholate (Thermo Fisher, 89904), 0.1 % SDS (w/v, Sigma, -250G), 50 mM Tris (Roth, 4855.2). The lysates (corresponding to 50 µg protein extract) were precipitated with acetone, digested to peptides and desalted as described in “Sample preparation for MS analysis”. The final desalting step was performed with 50 µl of a buffer solution of 80% ACN and 0.1% TFA. Prior to phosphopeptide enrichment, samples were filled up to 210 µl with 80% ACN and 0.1% TFA buffer solution. Phosphorylated peptides were enriched with Fe(III)-NTA cartridges (Agilent Technologies G 5496-60085) using the AssayMAP Bravo Platform (Agilent Technologies), as described in ^2^. Briefly, Fe(III)-NTA cartridges were washed with 250 μl 100% ACN/0.1% TFA and conditioned with 250 μl loading buffer consisting of 80% ACN and 0.1% TFA. After loading the samples into the cartridge, the columns were washed with 250 μl of loading buffer and then the phosphopeptides were eluted with 25 μl 1% ammonia directly into 25 μl 10% FA in water. Samples were dried using a speed vacuum centrifuge and stored at −20 °C until MS analysis.

**Data Independent Acquisition for enriched phosphopeptides**

Elutions from phosphopeptide enrichment or flowthroughs (used for whole proteome analysis) were reconstituted in 5% ACN, 95% Milli-Q water, with 0.1% FA and spiked with iRT peptides (Biognosys, Switzerland). Peptides were separated in trap/elute mode using the nanoAcquity M-Class Ultra-High-Performance Liquid Chromatography system (Waters, Waters Corporation, Milford, MA, USA) equipped with a trapping (Waters nanoEase M/Z Symmetry C18, 5μm, 180 μm x 20 mm) and an analytical column (Waters nanoEase M/Z Peptide C18, 1.7μm, 75μm x 250mm). Solvent A was water and 0.1% formic acid, and solvent B was acetonitrile and 0.1% formic acid. Between 40 and 80% of the samples were loaded onto the trapping column with a constant flow of solvent A at 5 μl/min. The trapping time was 6 minutes. Peptides were eluted over the analytical column at a constant flow of 0.3 μl/min. During the elution step, the percentage of solvent B increased nonlinearly from 0–40% in 60 min for phosphopetide analysis and in 120 min for whole proteome analysis. For MS analysis, the LC was coupled to an Exploris 480 (Thermo Fisher Scientific, Bremen, Germany) mass spectrometer using the Proxeon nanospray source. Peptides were introduced into the mass spectrometer via a Pico-Tip Emitter 360-μm outer diameter × 20-μm inner diameter, 10-μm tip (New Objective) heated at 300 °C, with a spray voltage of 2.2 kV applied. The capillary temperature was set at 300°C. The radio frequency (RF) was set to 30%.

For analysis of the whole proteome flowthroughs in DIA mode, full-scan MS spectra with a mass range of 350–1650 m/z were acquired in profile mode in Orbitrap with a resolution of 120,000 FWHM. The default charge state was set to 3+. The filling time was set to a maximum of 60 ms with a limit of 3 × 10^6^ ions. DIA scans were acquired with 40 mass window segments of differing widths over the MS1 mass range. Higher collisional dissociation fragmentation (stepped normalized collision energy; 25.5, 27, and 30%) was applied, and MS/MS spectra were acquired at a resolution of 30,000 FWHM with a fixed first mass of 200 m/z after accumulation of 3 × 10^6^ ions or after the filling time of 35 ms (whichever came first).

For the analysis of the phosphopeptide enrichment fraction, MS full scan spectra with a mass range of 350–1650 m/z were acquired in profile mode in Orbitrap with a resolution of 120,000 FWHM. The default charge state was set to 3+. The filling time was set to a maximum of 60 ms with a limit of 3 × 10^6^ ions. DIA scans were acquired with 30 mass window segments of differing widths over the MS1 mass range. Higher collisional dissociation fragmentation (stepped normalized collision energy; 25.5, 27, and 30%) was applied, and MS/MS spectra were acquired at a resolution of 30,000 FWHM with a fixed first mass of 200 m/z after accumulation of 3 × 10^6^ ions or after the filling time of 47 ms (whichever came first). Data were acquired in profile mode. Xcalibur 4.4 (Thermo Fisher) and Orbitrap Exploris 480 Tune version 2.0 were used for data acquisition and raw data processing.

**QUANTIFICATION AND STATISTICAL ANALYSIS**

**Data processing for TMT-labelled samples**

TMT-10plex data were processed using Proteome Discoverer v2.0 (Thermo Fisher). Data were searched against the relevant species-specific Fasta database (Uniprot database, Swissprot entry only, release 2016_01 for *Mus musculus,* with 16748 protein entries), using Mascot v2.5.1 (Matrix Science) with the following settings: the enzyme was set to trypsin, with up to 1 missed cleavage. MS1 mass tolerance was set to 10 ppm and for MS2 to 0.5 Da. Carbamidomethyl cysteine was set as a fixed modification and oxidation of Methionine as variable. Other modifications included the TMT-10plex modification from the quantification method used. The quantification method was set for quantification of reporter ions using HCD and MS3 (mass tolerance, 10 ppm). The false discovery rate for peptide-spectrum matches (PSMs) was set to 0.01 using Percolator ^3^.

Reporter ion intensity values for PSMs were exported and processed using procedures written in R (v. 3.4.1) as described in ^4^ . Briefly, PSMs mapping to reverse or contaminant hits, or having a Mascot score of less than 15, or reporter ion intensities of less than 1 x 10^3^ in all relevant TMT channels were discarded. The intensities of the TMT channels of the retained PSMs were then log2- transformed, normalized, and summarized into protein group quantities by taking the median value. At least two unique peptides per protein were required for identification, and only peptides with a missing value across all 10 channels were considered for quantification. Protein differential expression was evaluated using the limma package ^5^. Differences in protein abundances were statistically determined using an unpaired Student’s t test moderated by the empirical Bayes method. P values were adjusted for multiple testing using the Benjamini-Hochberg method (FDR, denoted as “adj. p”) ^6^. Proteins with *adj. p-value* < 0.25 were considered significantly affected unless otherwise stated.

**Data processing for samples with Data Independent Acquisition (DIA)**

Raw data from phosphopeptide enrichment elutions and flowthroughs (whole proteome) were analyzed using the directDIA pipeline in Spectronaut Professional v.15 (Biognosys AG). Data were searched against a species specific (Uniprot database, Swissprot entry only, release 2016_01 for *Mus musculus,* with 16748 protein entries) and a common contaminants database. Data were searched with the following modifications: Carbamidomethyl (C) (fixed) and Oxidation (M), Acetyl (Protein N-term), Phospho (STY) (variable, only for phosphopeptide enrichment elutions). PTM localization probability was set to 0.75. A maximum of 2 missed cleavages for trypsin and 5 variable modifications were allowed. Identifications were filtered to achieve an FDR of 1 % at the peptide and protein levels.

For phosphopeptide enrichment flowthroughs (whole proteome), relative quantification was performed in Spectronaut for each pairwise comparison using replicate samples from each condition with default settings, except: Major Group Quantity = median peptide quantity; Major Group Top N = OFF; Minor Group Quantity = median precursor quantity; Minor Group Top N = OFF; Data Filtering = Q value; Normalization Strategy = Local normalization; Row Selection = Automatic; Exclude Single Hit Proteins = TRUE. Tests for differential abundance were performed using an un-paired t-test between replicates. P values were corrected for multiple testing using the method described by Storey ^7^ to obtain false discovery rate adjusted p values (q values). Proteins with q-value < 0.05 and absolute log2 fold change > 0.58 were considered significantly affected unless otherwise stated.

For phosphopeptide enrichment elutions, peptide report table was exported from Spectronaut and further processed with R (v.3.6.3) and R studio server (v. 1.2.5042) using in-house pipelines and scripts. Phosphosite-level intensities were determined by summing the intensities of all precursors containing a given phosphosite. Phosphosite intensities were log2-transformed and normalized by quantile normalization using the preprocessCore library ^8^. Differences in phosphosite levels were determined statistically using a paired Student’s t-test moderated by the empirical Bayes method as implemented in the limma package ^5^. False discovery rate was estimated using *fdrtool* ^9^. The PhosR package ^10^ with default settings was used to identify the most highly regulated kinases and the corresponding regulated top phosphosites.

**Analysis of cellular compartments**

Normalized protein intensities obtained from proteomic data were analysed using Compartment Normalized Value analysis, as described in Parca et al. ^11^. Cellular compartments were defined using the following Gene Ontology Cellular Component terms and their children terms:

| **Compartment** | **GO term** |
| --- | --- |
| Nucleus | GO:0005634 |
| Cytosol | GO:0005737 |
| Mitochondrion | GO:0005739 |
| Extracellular | GO:0005576, GO:0031012, GO:0044421, GO:0044420 |
| Peroxisome | GO:0005777 |
| Lysosome | GO:0005764 |
| ER | GO:0005783 |
| Golgi | GO:0005794 |
| Membrane | GO:0005886 |
| Cytoskeleton | GO:0005856 |

**Quantification and localization of lactadherin from mouse LV sections**

The images of stained cryosections of LV mice, obtained in high-content fluorescence microscope, were used for quantification and localization of lactadherin. QuPath software (version 0.30.) was used for this purpose. The total area of the vessels (endothelial cell area + smooth muscle cell area) was measured with the Wang tool of the software, and vessels were classified into 4 categories based on area range: >1000 μ^2^, 1000 – 500 μ^2^, 500-100 μ^2^; and <100 μ^2^. The positive area of lactadherin was measured within each vessel the Wang tool of the software, normalized to the total area of the vessel, and described as the percentage of positive lactadherin.

The localization of lactadherin within each vessel was assessed directly by visualization of lactadherin-positive areas and described as “Smooth Muscle cells” or “Interface of Endothelial Cells and Muscle cells” when lactadherin was located in the muscle cell area region of, or in the interface between muscle cells and endothelial cells, respectively.

References:

1. Silva AC, Rodrigues SC, Caldeira J, Nunes AM, Sampaio-Pinto V, Resende TP, Oliveira MJ, Barbosa MA, Thorsteinsdottir S, Nascimento DS, Pinto-do OP. Three-dimensional scaffolds of fetal decellularized hearts exhibit enhanced potential to support cardiac cells in comparison to the adult. *Biomaterials* 2016;**104**:52-64.

2. Post H, Penning R, Fitzpatrick MA, Garrigues LB, Wu W, MacGillavry HD, Hoogenraad CC, Heck AJ, Altelaar AF. Robust, Sensitive, and Automated Phosphopeptide Enrichment Optimized for Low Sample Amounts Applied to Primary Hippocampal Neurons. *J Proteome Res* 2017;**16**:728-737.

3. Brosch M, Yu L, Hubbard T, Choudhary J. Accurate and sensitive peptide identification with Mascot Percolator. *Journal of proteome research* 2009;**8**:3176-3181.

4. Heinze I, Bens M, Calzia E, Holtze S, Dakhovnik O, Sahm A, Kirkpatrick JM, Szafranski K, Romanov N, Sama SN, Holzer K, Singer S, Ermolaeva M, Platzer M, Hildebrandt T, Ori A. Species comparison of liver proteomes reveals links to naked mole-rat longevity and human aging. *BMC biology* 2018;**16**:82.

5. Ritchie ME, Phipson B, Wu D, Hu Y, Law CW, Shi W, Smyth GK. limma powers differential expression analyses for RNA-sequencing and microarray studies. *Nucleic acids research* 2015;**43**:e47.

6. Benjamini Y, Hochberg Y. Controlling The False Discovery Rate - A Practical And Powerful Approach To Multiple Testing. *J Royal Statist Soc, Series B* 1995;**57**:289-300.

7. Storey JD. A direct approach to false discovery rates. 2002;**64**:479-498.

8. Bolstad BM, Irizarry RA, Astrand M, Speed TP. A comparison of normalization methods for high density oligonucleotide array data based on variance and bias. *Bioinformatics* 2003;**19**:185-193.

9. Strimmer K. A unified approach to false discovery rate estimation. *BMC Bioinformatics* 2008;**9**:303.

10. Kim HJ, Kim T, Hoffman NJ, Xiao D, James DE, Humphrey SJ, Yang P. PhosR enables processing and functional analysis of phosphoproteomic data. *Cell Rep* 2021;**34**:108771.

11. Parca L, Beck M, Bork P, Ori A. Quantifying compartment-associated variations of protein abundance in proteomics data. *Molecular systems biology* 2018;**14**:e8131.

**2. Supplementary figures**


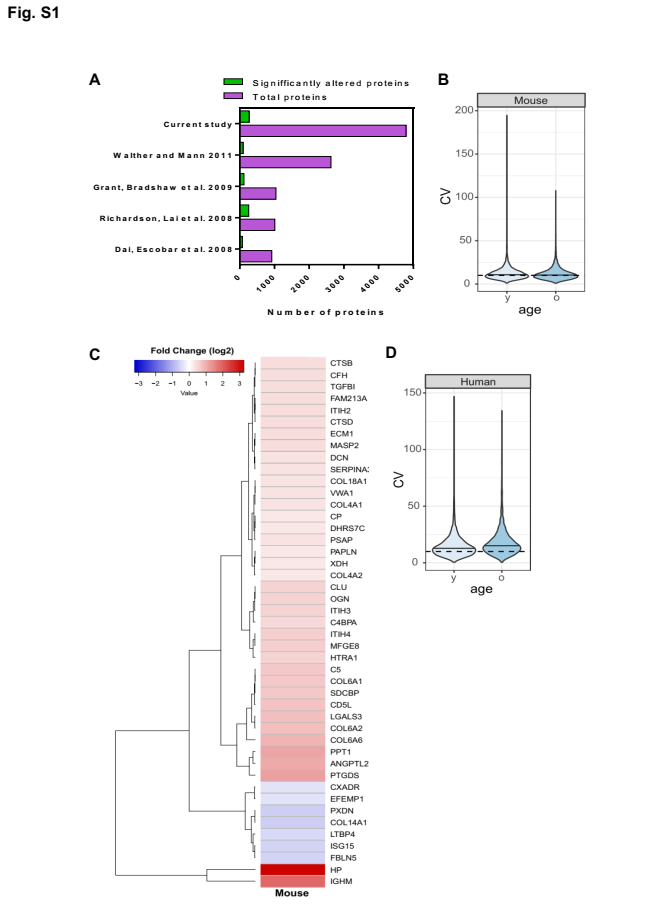


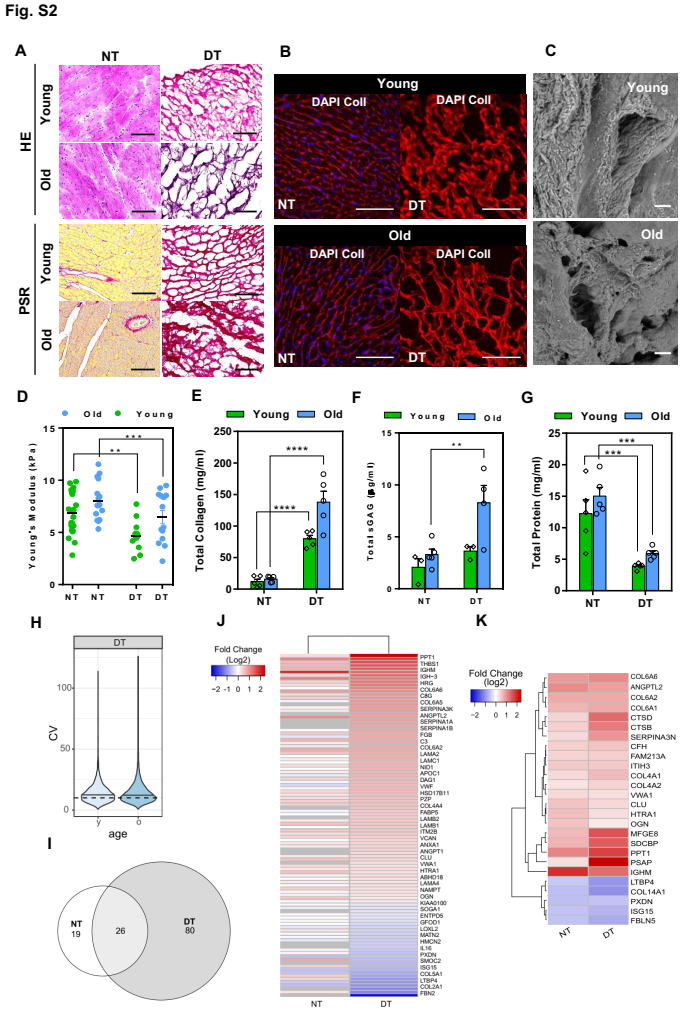


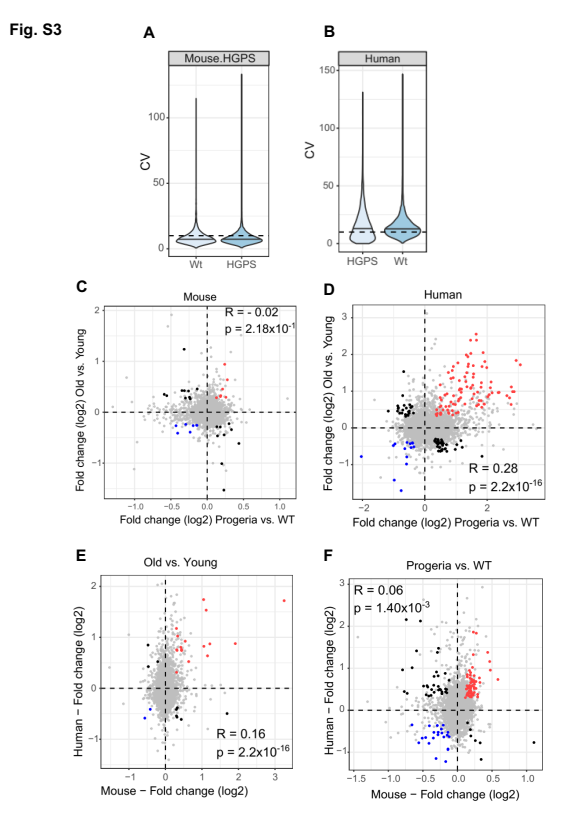


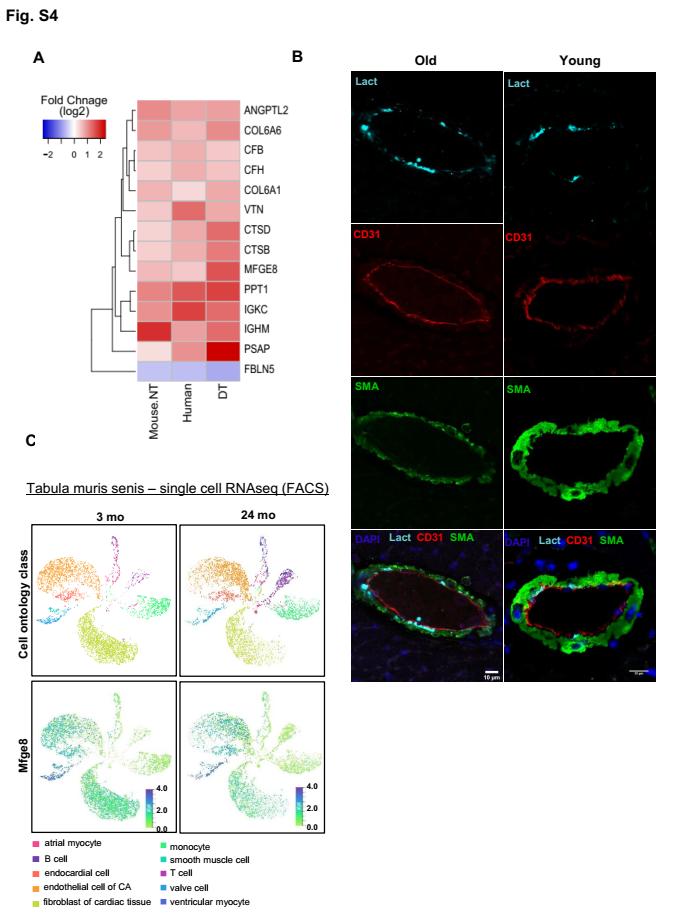


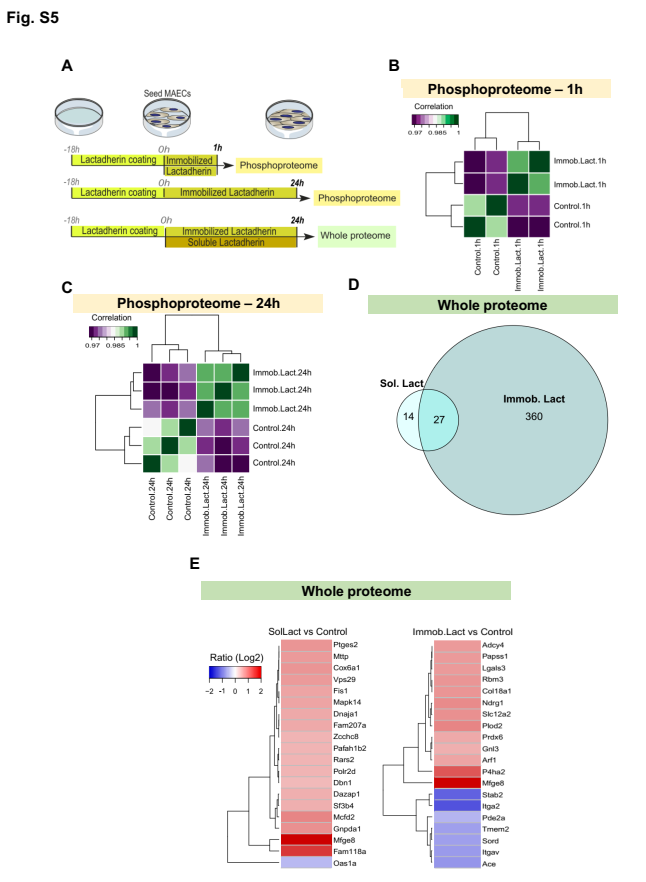


**Figure S1. Aging effect on cardiac proteome of mouse and human left ventricle (LV).** *A*, Bar plot showing coverage of previous studies compared with the current study. *B***,** Violin plot showing the coefficient of variation for all quantified proteins in two age groups in mice. “y”, young; “o”, old group; n= 5, per group. *C*, Heatmap displaying hierarchical clustering based on Pearson correlation of all quantified proteins by TMT-based quantitative mass spectrometry of mouse LV tissues of five young and old LV mice. *D,* Violin plot showing the coefficient of variation for all quantified proteins between two human age groups. y”, young; “o”, old group; n= 5, per group.

**Figure S2. Decellularization of LV mouse heart tissue.** *A*, Hematoxylin-Eosin (HE), Picro Sirius Red (PSR) staining of NT and DT from LV tissues of young and old mice. Scale bar, 100 mm. *B*, Immunofluorescence for collagen I (ColI) (red) and nuclear DAPI (blue) of NT and DT from ventricular tissues of young and old mice. Scale bar, 100 μm. *C*, Scanning electron micrographs of DT from young and old mice. Scale bar, 200 μm. *D*, Elasticity of NT and DT from young and old LV mouse tissues measured by an atomic force microscopy indentation technique. Biochemical quantifications of total (*E*) collagen, (*F*) sulfated glicosaminoglycans (GAGs) and (*G*) protein of NT and DT from young and old LV mouse tissue. Results are Average ± SEM, and symbols in bars correspond to the number of mice analyzed (n), (*B*) and (*C*): n= 5, per group (young and old), (*D*): young, n= 3 and old, n=4. ****: p < 0.0001; ***: p < 0.001; **: p < 0.01, two-way ANOVA. *H*, Violin plot showing the coefficient of variation for all quantified proteins across two age groups in DT of mice LV. n=5, per group; “y”, young; “o”, old mice LV. *I*, Venn diagram showing the number of significantly altered ECM proteins (adj.p<0.25 and FC > 0.58) in NT and DT. It shows the subset of overlapping proteins and also the proteins that were exclusively quantified in each dataset. *J*, Heatmap displaying several ECM proteins identified in DT that were not observed in whole cell lysates (NT). *K*, Heatmap showing the common ECM proteins most affected by age in NT and DT (adj.p<0.25).

**Figure S3 – HGPS affects the proteome of mouse and human left ventricle (LV).** *A*, Violin plot showing the coefficient of variation (CV) for all quantified proteins between two mouse groups, progeroid (HGPS) and wild-type (wt). *B*, Violin plot displaying the coefficient of variation (CV) for all quantified proteins between human groups, progeroid group (HGPS) and non-progeroid group (Wt). Scatter plots showing fold changes in (*C*) mice and (*D*) humans between chronological aging (old and young (y-axis)) and pathological aging (progeria and wild-type (x-axis)). Scatter plots showing fold changes in (*E*) chronological aging (old vs. young) and (*F*) pathological aging (progeria vs. WT) between mouse (x-axis) and human (y-axis). R corresponds to Pearson’s correlation coefficient; p corresponds to p-value.

**Figure S4 – ECM proteins commonly altered in proteome datasets, expression and localization of lactadherin in LV mouse tissue during aging.** *A*, Heatmap showing fold change (log2) for all common altered ECM proteins (p<0.05) in 3 of 4 analysed proteomic datasets (NT, Human and DT). *B*, Representative images of immunofluorescence staining for lactadherin (Lact) (cyan), CD31 (red), smooth muscle actin (SMA) (green), and DAPI (blue) from LV sections of 3 months (young) and 22 months old (old) mice. Scale bar: 10 μm**.** *C*, Single-cell transcriptomics analysis of Tabula muris senis (Pisco et al., 2019), showing analysis of Mfge8 (lactadherin) expression levels at 3 and 24 months of age (right and left panel, respectively) in atrial myocytes (pink), B cells (purple), endocardial cells (orange), endothelial cells of coronary artery (CA) (light orange), fibroblasts (light green), monocytes (turquoise), smooth muscle cells (dark turquoise), T cells (dark pink), valve cells (light blue) and ventricular myocytes (dark blue) in the heart.

**Figure S5. Effect of soluble and immobilized lactadherin in MAECs.** A, Schematic representation of the experimental setup; 10μg/ml and 500ng/ml of lactadherin were used for immobilized and soluble stimuli, respectively. The phosphoproteome was analysed at 1 hour and 24 hours using immobilized lactadherin only. In contrast, the whole proteome in MAECs was analysed only after 24 hours, but MAECs were stimulated by immobilized and soluble lactadherin. *B*, *C* Heatmaps showing correlation within each experimental group: (*B*) 1 hour and (*C*) 24 hours after stimulation of MAECs with immobilized lactadherin. The reproducibility of the experiments was shown by the high correlation value within each experimental group. (*D*) Venn diagram showing the number of altered proteins (p<0.05 and FC > 0.58) in the whole proteome of MAECs after 24 hours stimulation with soluble or immobilized lactadherin**.** (*E*) The top 20 proteins (FC > 0.58 and ranked by lowest p-value) that were affected by soluble (SolLact) or immobilized (Immob.Lact) lactadherin in the whole proteome of MAECs are shown in the left and right heatmaps, respectively. Lactadherin/Mfge8 was shown to be a highly affected protein, but this probably indicates contamination by the recombinant protein used in the experimental procedure.
